# Spillover, hybridization, and persistence in schistosome transmission dynamics at the human–animal interface

**DOI:** 10.1073/pnas.2110711118

**Published:** 2021-10-06

**Authors:** Anna Borlase, James W. Rudge, Elsa Léger, Nicolas D. Diouf, Cheikh B. Fall, Samba D. Diop, Stefano Catalano, Mariama Sène, Joanne P. Webster

**Affiliations:** ^a^Centre for Emerging, Endemic and Exotic Diseases, Department of Pathobiology and Population Sciences, Royal Veterinary College, University of London, Hatfield AL9 7TA, United Kingdom;; ^b^Communicable Diseases Policy Research Group, Department of Global Health and Development, London School of Hygiene and Tropical Medicine, London WC1E 7HT, United Kingdom;; ^c^Faculty of Public Health, Mahidol University, Nakhon Pathom 73170, Thailand;; ^d^Institut Supérieur de Formation Agricole et Rurale, Université de Thiès, Bambey BP 54, Senegal;; ^e^Unité de Formation et de Recherche des Sciences Agronomiques, d’Aquaculture et de Technologies Alimentaires, Université Gaston Berger, BP 32000 Saint-Louis, Senegal;; ^f^Service de Parasitologie – Mycologie, Faculté de Médecine, Pharmacie et Odontologie, Université Cheikh Anta Diop, BP 5005 Dakar, Senegal

**Keywords:** schistosomiasis, hybrids, modelling, *R*_*0*_, spillover

## Abstract

Zoonotic spillover and hybridization of parasites are major emerging public and veterinary health concerns at the interface of infectious disease biology, evolution, and control. Schistosomiasis is a neglected tropical disease of global importance caused by parasites of the *Schistosoma* genus, and the *Schistosoma* spp. system within Africa represents a key example of a system where spillover of animal parasites into human populations has enabled formation of hybrids. Combining model-based approaches and analyses of parasitological, molecular, and epidemiological data from northern Senegal, a region with a high prevalence of schistosome hybrids, we aimed to unravel the transmission dynamics of this complex multihost, multiparasite system. Using Bayesian methods and by estimating the basic reproduction number (*R_0_*), we evaluate the frequency of zoonotic spillover of *Schistosoma bovis* from livestock and the potential for onward transmission of hybrid *S. bovis* × *S. haematobium* offspring within human populations. We estimate *R_0_* of hybrid schistosomes to be greater than the critical threshold of one (1.76; 95% CI 1.59 to 1.99), demonstrating the potential for hybridization to facilitate spread and establishment of schistosomiasis beyond its original geographical boundaries. We estimate *R_0_* for *S. bovis* to be greater than one in cattle (1.43; 95% CI 1.24 to 1.85) but not in other ruminants, confirming cattle as the primary zoonotic reservoir. Through longitudinal simulations, we also show that where *S. bovis* and *S. haematobium* are coendemic (in livestock and humans respectively), the relative importance of zoonotic transmission is predicted to increase as the disease in humans nears elimination.

## Significance

The threat to public health that is presented by zoonotic spillover of pathogens from animal reservoirs is predicted to increase with rapid anthropogenic changes and global trends such as migration and changing land use. Schistosomiasis currently infects more than 220 million people worldwide, and the multihost *Schistosoma* spp. system within Africa is a key example of where spillover of animal parasites into human populations has enabled the formation of viable hybrid parasite genotypes. Our study demonstrates how zoonotic spillover and complex interactions between pathogen species, such as parasite hybridization, may have implications such as resilience to current disease control strategies and may facilitate the spread of tropical diseases such as schistosomiasis beyond their original geographical boundaries.

There is a growing acknowledgment of the profound threat that zoonoses (diseases transmitted between animals and humans) pose to human health worldwide, with animal reservoirs presenting diverse complications to elimination efforts for many existing diseases, as well as novel threats in the form of newly emerging diseases (1, 2). Global trends such as increased migration, altering agricultural practices, and a changing climate are all predicted to enhance the potential for human and animal populations to encounter new infectious agents, thereby also increasing opportunities for coinfection by multiple pathogen species within the same host (3–7). Such mixed infections can lead to exchange of genetic material between the coinfecting agents, generating new pathogen genotypes. In the case of helminth parasites which sexually reproduce, this can occur through heterospecific (between-species) mate pairings (8, 9), which can lead to the formation of hybrid offspring (9, 10). Hybridizations, as well as subsequent introgressions (the introduction of single genes or chromosomal regions from one species into another through repeated backcrossing), represent an additional source of genetic variation that may drive parasite evolution, with potential implications including increased host and geographical range, altered pathology, resistance to drug therapy, and, ultimately, persistence in the face of elimination efforts (9). The presence of interspecific hybridizations and introgressions brings an increased level of complexity to disentangling the transmission dynamics of multihost systems, requiring multimodal and original approaches and necessitating data from across disciplines and scales—from the molecular to the population level (11).

The significance of zoonotic transmission and parasite hybridization is exemplified by the case of schistosomiasis. Schistosomiasis is a neglected tropical disease estimated to infect over 220 million people, over 90% of whom reside in sub-Saharan Africa (12–14). The disease is caused by parasites of the *Schistosoma* genus, which have a complex life cycle that includes sexual reproduction in a mammalian definitive host and indirect transmission via a freshwater snail intermediate host. The schistosome species *Schistosoma haematobium* (the causative agent for urogenital schistosomiasis in people) has been demonstrated to form viable hybrids and introgressions with closely related schistosome species of the larger Haematobium group which infect livestock, notably *Schistosoma bovis*, *Schistosoma curassoni*, and *Schistosoma mattheei* (causative agents of intestinal schistosomiasis in livestock) (15–20). Hybrids between *S. haematobium* and *S. bovis* have been reported in human patients across sub-Saharan Africa, including Senegal, Niger, Côte d’Ivoire, and Mali (21–23), as well as in the recent outbreaks of schistosomiasis in Corsica (24, 25). One recent study in northern Senegal, a region where both *S. haematobium* and *S. bovis* are coendemic, indicated that ongoing pairing between these two species leads to creation of hybrids in human hosts and is occurring here via zoonotic spillover of *S. bovis* from a livestock reservoir to people who are simultaneously infected with *S. haematobium* (19). Spillover is defined as transmission, from a reservoir to a defined target host, of a pathogen that cannot normally be sustained within the target host population (26, 27), while reservoirs are defined as one or more populations in which a pathogen can be maintained and from which infection is transmitted to a defined target population (28). While molecular data from the study in Senegal indicated that *S. bovis* cannot be maintained by the human population (hence fitting the definition for a spillover pathogen), onward transmission of hybrids was shown to occur within the human population via backcrossing and introgressions, leading to a complex array of observed miracidia genotypes (miracidia being the first larval schistosome stage which hatches from eggs shed by an infected host) from human specimens.

The public health impact of zoonotic spillover is fundamentally determined by the force of infection from the reservoir species and the potential for onward transmission and persistence within the human population (29), with identification of key hosts and reservoirs also critical to our understanding of multihost systems. These aspects of transmission are crucial for determining appropriate targets for interventions, and ultimately the feasibility of disease elimination, yet are often little-studied and poorly understood.

Given that formation of F1 hybrids requires a host to be simultaneously infected with *S. haematobium* and *S. bovis,* attention is naturally focused on geographic localities where circulation of these two closely related schistosome species may be sympatric. It has remained unclear whether humans can act as maintenance hosts for these hybrid parasites after they have been generated by the initial cross-species pairing, or if ongoing zoonotic spillover is required for hybrids to persist in the human population. The fact that hybrid schistosomes identified in the Corsican outbreak were found to have been imported from Senegal (25) highlights both the need to understand the zoonotic potential of *S. bovis* and, crucially, to evaluate the potential for hybridization to facilitate spread and establishment of schistosomiasis beyond its original geographical boundaries.

Here we combine mathematical modeling and statistical approaches with molecular and epidemiological data from a recent study in northern Senegal (19) in order to evaluate the spillover and multihost dynamics within the Haematobium group hybrid schistosome system. We examine the relative importance of zoonotic spillover in maintaining transmission both at current levels of endemicity and in scenarios where the human disease may be nearing elimination and characterize the relative role of the livestock species involved in zoonotic transmission. Model structure was informed by the insights from the molecular data and parameterized using Bayesian approaches, with output then applied to existing frameworks for classifying multihost systems and zoonotic disease threats (26, 27, 29). This included estimation of the basic reproduction number, or *R*_*0,*_ within each host species. Given the lack of literature on key aspects of *S. bovis* biology necessary for fitting the model to observable data, we also describe a Bayesian approach for inferring the relationship between worm burden and fecal egg count using postmortem studies of naturally occurring infections. We explore the potential for density-dependent effects in this relationship and consider the implications of such effects for macroparasite transmission dynamics in general.

With current trends in anthropogenic activities predicted to drive infectious disease emergence at the global scale, integrative methods for characterizing pathogen transmission events at the animal–human interface and evaluating the consequences of novel pathogen interactions, including hybridizations, have been identified as key scientific challenges (9, 30, 31). As we seek to understand increasingly complex disease dynamics, our work will therefore be of relevance not only for the control of schistosomiasis but also for a growing number of disease systems in our rapidly changing world.

## Results

**Fig. 1.**
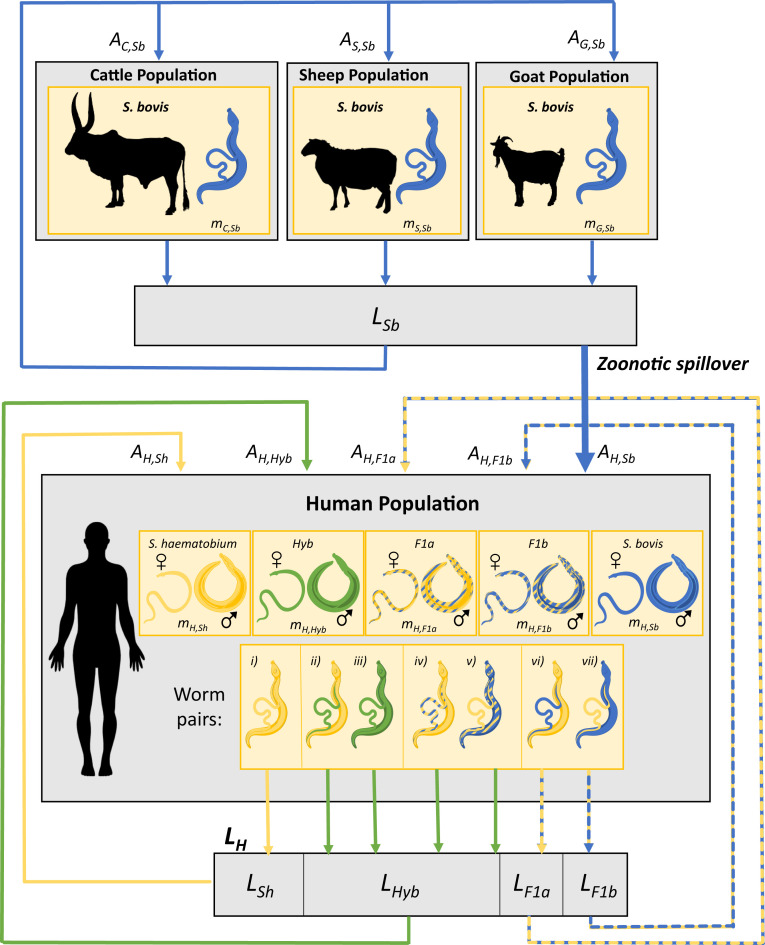
Schematic of the multihost, multiparasite transmission model: *S. haematobium*, *S. bovis*, and Haematobium group hybrids in human and livestock populations. Parameter description and categorization of worm genotypes in definitive hosts are detailed in *SI Appendix*, Tables S1 and S2.

The framework for the mathematical model (summarized in the schematic shown in Fig. 1) was based on the multihost, multiparasite transmission cycle for Haematobium group schistosomes proposed in ref. 19, which was informed by molecular and epidemiological findings from surveys of human, snail, and livestock populations. Key aspects of the system which are incorporated into the model include zoonotic transmission of *S. bovis* from the livestock reservoir to the human population, interactions between worm genotypes including formation of F1 *S. bovis* × *S. haematobium* hybrids in the human host via bidirectional cross-species pairings, and onward transmission of hybrid genotypes via backcrossing of F1 worms.

Definitive hosts are humans (*H*), cattle (*C*), sheep (*S*), and goats (*G*), (*d* ∈{*H*, *C*, *S*, *G*}). Here, livestock can be infected with *S. bovis*, and humans can be infected with five categories of worm genotypes: *S. haematobium* (*Sh*), *S. bovis* (*Sb*), two categories of first-generation or F1 hybrids (*F1a* being the product of a pairing between a male *S. haematobium* and a female *S. bovis*; *F1b* being the product of a pairing between a female *S. haematobium* and a male *S. bovis*), and later-generation/introgressed *S. haematobium* × *S. bovi*s hybrids (*Hyb*).

The model tracks the mean number of worms (*m*_*d,j*_) of each of these schistosome genotypes *j* (where *j* ∈{*Sh*, *Sb*, *F1a*, *F1b*, *Hyb*}), within definitive host population *d.* In longitudinal simulations it is assumed that praziquantel (the drug used for mass drug administration, which is the mainstay of schistosomiasis control efforts) is equally efficacious against all worm genotypes.

### Spillover Dynamics.

The median posterior estimates of the mean worm burden for each schistosome genotype in the human population (*m*_*H,i*_) are given in Table 1, together with the estimated transmission rates from the larval pool to the human population. Confidence intervals are given as 95% Bayesian credible intervals (BCI).

**Table 1.** Estimated mean worm burden in the human population (*m*_*H,j*_) of each distinguished genotype (*j*) and estimated transmission rate from larval pool *L*_*j*_ to human population (*A*_*H,j*_)

Worm genotype (*j*)	Estimated mean worm burden in the human population *m*_*H,j*_, median posterior (95% BCI)	Median estimated percentage of all worm genotypes (95% BCI)	Transmission rate from larval pool *L*_*j*_ to human population *A*_*H,j*_ (days) median posterior (95% BCI)
*Sh* (*Schistosoma haematobium*)	28.44 (23.33, 35.01)	73.1% (70.29,75.74)	*A*_*H,Sh*_ = 0.09 (BCI:0.08, 0.11)
*Hyb* (later-generation hybrids: product of later backcrosses)	10.11 (8.06, 12.76)	25.83% (23.06, 28.46)	*A*_*H,Hyb*_ = 0.09 (BCI:0.07, 0.10)
*F1a* (first-generation product of pairing between male *S. haematobium* and female *S. bovis*)	0.12 (0.06, 0.22)	0.3% (0.15,0.53)	*A*_*H,F1a*_ = 0.17 (BCI: 0.06,0.53)
*F1b* (first-generation product of pairing between female *S. haematobium* and male *S. bovis*)	0.21 (0.12, 0.35)	0.55% (0.31,0.83)	*A*_*H,F1b*_ = 0.19 (BCI: 0.07,0.55)
*Sb* (*S. bovis*, transmitted to human population via spillover from livestock reservoir)	0.07 (0.03, 0.14)	0.17% (0.07, 0.34)	*A*_*H,Sb*_ = 8.3 × 10^−4^ (BCI: 3.2 × 10^−4^, 1.8 × 10^−3^)

Data from the 2016 survey of school-aged children in northern Senegal (*n* = 375).

Estimates indicate that the majority of overall worm burden in human hosts is composed of *S. haematobium* (median estimated percentage of worm burden 73.1%; 95% BCI 70.3 to 75.7%) and the majority of the hybrid worm burden are the later-generation/introgressed *Hyb* genotype (median 96.76%; 95% BCI 95.32 to 97.88%).

The estimated mean worm burden of *S. bovis* in humans (0.07; 95% BCI 0.03 to 0.14) corresponds to a very low percentage (0.17%; 95% BCI 0.07 to 0.34%) of the total Haematobium group worm burden in the human population being zoonotically acquired and, accordingly, the median estimated transmission rate from the *S. bovis* larval pool to the human population (*A*_*H,Sb*_; Table 1) was several orders of magnitude lower than the corresponding transmission rates for other genotypes. Here, transmission rates from the larval pool to humans were estimated to be similar for all genotypes derived from miracidia shed by the human population (*A*_*H,Sh*_, *A*_*H,Hyb,*_, *A*_*H,F1a*_, and *A*_*H,F1b*_), noting wider CIs for *A*_*H,F1a*_ and *A*_*H,F1b*_ due to the lower number of miracidia these estimates were based on (19).

### Estimation of Basic Reproduction Number for Hybrid Schistosomes (*R*_*0*_^*H,Hyb*^).

In this framework the basic reproduction number, *R*_*0*_*,* is defined as the average number of mated female offspring produced by one mated adult female schistosome in the absence of constraints on population growth (32). The estimation of *R*_*0*_ can therefore be used to evaluate the capacity of *S. haematobium* × *S. bovis* hybrids to be maintained in the human population in the absence of zoonotic spillover. If the average *Hyb* female does not replace herself in the next generation (*R*_*0*_^*H,Hyb*^ < 1), this genotype category could not be maintained without the creation of novel hybrids via zoonotic spillover.

The basic reproduction number for the hybrid genotype *Hyb* (*R*_*0*_^*H,Hyb*^) within the human population was estimated to be 1.76 (95% BCI 1.59 to 1.99; Table 2), comparable to the value estimated for *S. haematobium* (1.90; 95% BCI 1.74 to 2.14).

**Table 2.** Estimated within-species basic reproductive number (*R*_*0*_) for Schistosome genotypes in human and livestock populations and estimated overall basic reproductive number for *S. bovis* under current conditions with all host species involved in transmission and in simulated conditions where transmission from a host population is eliminated

	Host species	Within-species *R*_*0*_ estimate (median, 95% CI)
Estimated *R*_*0*_ within host species (*R*_*0*_^*d,j*^)		
Schistosome genotype		
* Hyb*	Human *R*_*0*_^*H,Hyb*^	1.76 (1.59,1.99)
* S. * *haematobium*	Human *R*_*0*_^*H,Sh*^	1.9 (1.74, 2.14)
* S. bovis*	Cattle (*R*_*0*_^*C,Sb*^)	1.43 (1.24,1.85)
* S. bovis*	Goats (*R*_*0*_^*G,Sb*^)	0.03 (0.02,0.05)
* S. bovis*	Sheep (*R*_*0*_^*S,Sb*^)	0.04 (0.02,0.08)
Overall *R*_*0*_ estimation for multihost *S. bovis* system; all host species and with removal of species simulated		
Worm genotype		
* S. bovis*	Cattle, sheep, and goats (*R*_*0*_^*tot,Sb*^)	1.49 (1.34,1.90)
* S. bovis*	Sheep and goats (cattle removed)	0.07 (0.04,0.12)
* S. bovis*	Cattle and sheep (goats removed)	1.47 (0.04,0.12)
* S. bovis*	Cattle and goats (sheep removed)	1.46 (1.28,1.87)

Values represent median estimates (and 95% CIs) derived from uncertainty analysis.

### Projected Impact of Removing Zoonotic Transmission: Longitudinal Simulations under Varying Treatment Coverage Levels.

**Fig. 2.**
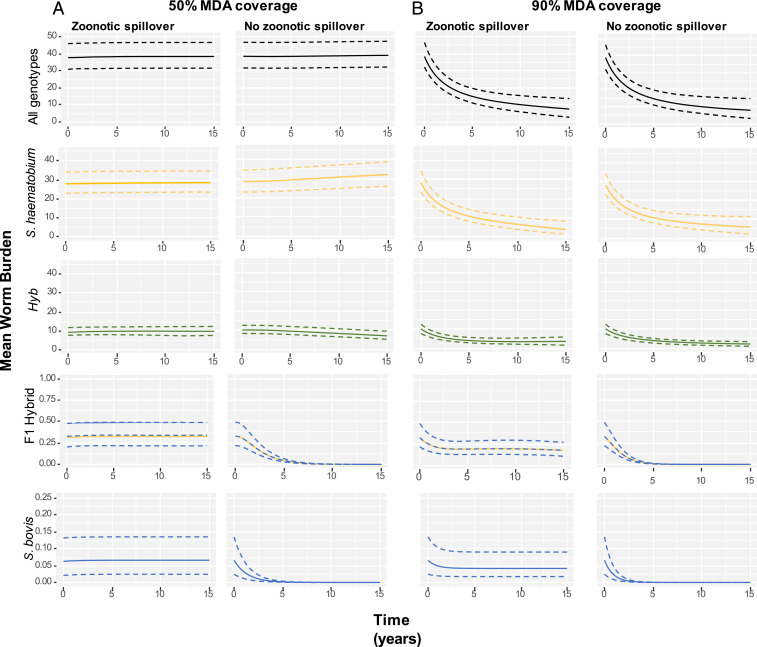
Longitudinal simulations under assumed current level of MDA with praziquantel (*A*: 50% annual coverage school-aged children) and under enhanced coverage (*B*: 90% annual coverage school-aged children). Mean worm burden in human population of *S. haematobium*, *Hyb*, F1 hybrids, and *S. bovis* under current level of zoonotic transmission and with no zoonotic transmission. Median shown as solid lines, dashed lines show 95% CIs.

At all treatment coverage levels simulated (50% and 90% shown in Fig. 2; 75% coverage, *SI Appendix*, Fig. S10) with no zoonotic transmission of *S. bovis*, transmission of the *Hyb* genotype was predicted to persist after 15 y (Fig. 2). This indicates that even under the increased pressure exerted by higher levels of mass drug administration (MDA) hybrids can be maintained within the human population without zoonotic transmission of *S. bovis* to the human population and generation of novel hybrids.

In the absence of zoonotic transmission, predicted mean burden of F1 hybrids and *S. bovis* declines rapidly to zero, as would be expected (Fig. 2). However, this is predicted to have minimal impact on the overall worm burden and lead to only a slight decrease in the burden of the later generation *Hyb* hybrids. At current treatment coverage levels (assumed 50% coverage of school-aged children based on World Health Organization preventative chemotherapy databank for Senegal; ref. 33) the overall mean worm burden after 15 simulated years was predicted to be 38.70 (95% CI 31.95 to 46.84) with zoonotic transmission and 39.12 in the absence of zoonotic transmission (95% CI 32.30 to 47.22), corresponding to a median 1% difference in worm burden between the two scenarios (95% CI 0.23 to 2.07%).

**Fig. 3.**
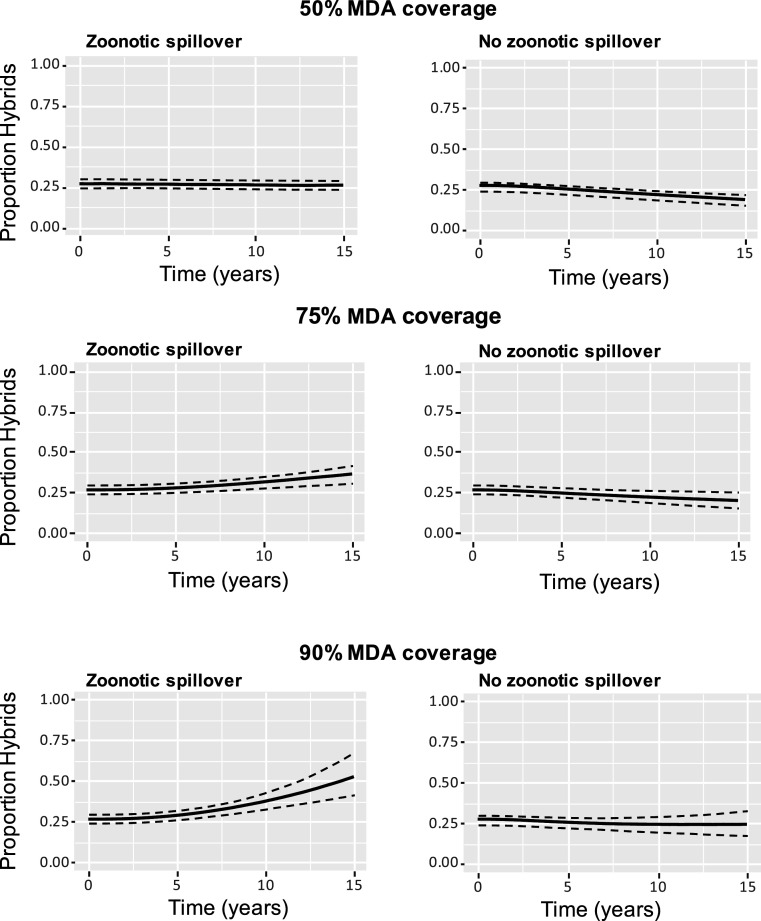
Longitudinal simulations: Predicted impact of varied MDA coverage on proportion of worm burden in the human population that are hybrids under current level of zoonotic transmission (*Left*) and with no zoonotic transmission (*Right*).

Although spillover of *S. bovis* was estimated to contribute a very small proportion to the overall worm burden in the human population at current levels of MDA, the relative importance of ongoing zoonotic transmission from a stable reservoir in terms of worm burden and genotype composition in the human population was predicted to increase considerably under scenarios of increasing MDA coverage. This is illustrated in Fig. 3, where it can be seen that, at current levels of zoonotic spillover under MDA coverage levels of 75% and 90%, the percentage of mean worm burden that is composed of hybrids would be predicted to increase. This effect was most marked at 90% treatment coverage, with the proportion of mean worm burden that is composed of hybrids predicted to reach more than 50% after 15 simulated years (median estimate 51.90%, 95% CI 41.17 to 67.36).

### Evaluating the Zoonotic *S. bovis* Reservoir and Identifying Key Hosts: Estimation of Within-Species and Overall Basic Reproduction Numbers (*R*_*0*_^*d,Sb*^ and *R*_*0*_^*tot,Sb*^).

In a multihost system the overall basic reproduction number (denoted *R*_*0*_^*tot*^) will be dependent on the basic reproduction number for each host species within that system and the level of transmission between species. The *R*_*0*_^*tot*^ must be greater than 1 for transmission of a pathogen to persist within a host community, meaning *R*_*0*_^*tot*^ <1 can be interpreted as the threshold for elimination.

Following existing frameworks for classifying multihost systems (27, 28), if only those hosts for which *R*_*0*_^*d*^ > 1 can be considered maintenance hosts (i.e., capable of independently sustaining transmission in the absence of other host species), then in this system only cattle can be considered maintenance hosts for *S. bovis* (Table 2) with median *R*_*0*_^*C,Sb*^ estimated as 1.49 (95% CI 1.34 to 1.90) and *R*_*0*_^*d,Sb*^ estimates well below 1 for goats (*R*_*0*_^*G,Sb*^ = 0.03, 95% CI 0.02 to 0.05) and sheep (*R*_*0*_^*S,Sb*^ = 0.04, 95% CI 0.02 to 0.08). Furthermore, if essential hosts are defined as those for which transmission cannot be sustained (*R*_*0*_^*tot*^ < 1) if their contribution to transmission is removed, then cattle can be considered essential hosts in this system, with median *R*_*0*_^*tot,Sb*^ in the absence of cattle estimated to be 0.07 (95% CI 0.04 to 0.12). In the absence of the contribution of sheep or goats, the median *R*_*0*_^*tot,Sb*^ estimates and 95% CIs remain greater than one.

### Impact of Targeting the Cattle Population on *S. bovis* Transmission within Livestock.

Given that removal of small ruminants was predicted to have minimal impact on *S. bovis* transmission dynamics in the study region (minimal change to *R*_*0*_^*tot,Sb*^ in their absence) only the impact of interventions targeting the cattle population were explored in the multihost model.

**Fig. 4.**
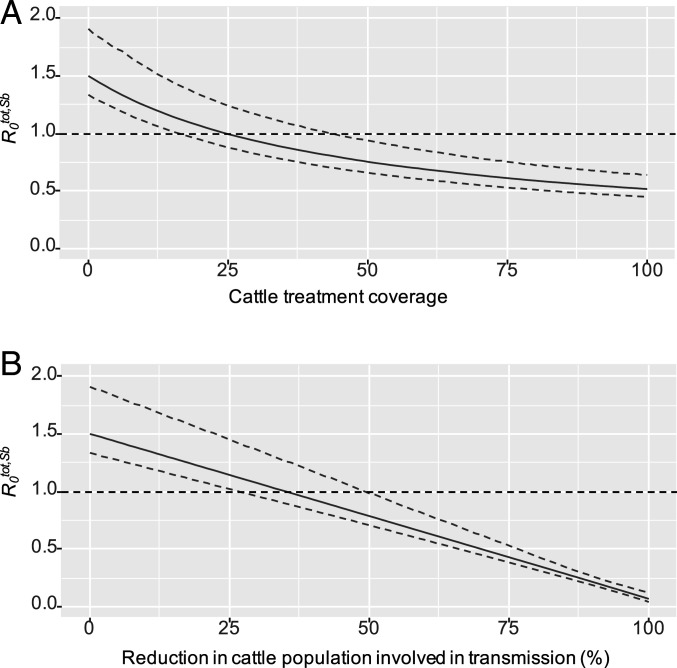
The predicted impact on overall basic reproductive number (*R*_*0*_^*tot,Sb*^) for *S. bovis* of (*A*) annual treatment of the cattle population and (*B*) reducing the proportion of the cattle population contributing to schistosomiasis transmission. Solid blue lines indicate the median estimates from the uncertainty analysis and dashed blue lines indicate the 95% CIs.

Treating cattle with a 95% efficacious treatment (34) is predicted to have a considerable impact on *S. bovis* transmission, reducing the *R*_*0*_^*tot,Sb*^ to less than one (Fig. 4*A*). There is, however, uncertainty around the treatment coverage necessary to reduce *R*_*0*_^*tot,Sb*^ below this threshold (median estimate 25%; 95% CI 17 to 44%). Alternatively, reducing the size of the cattle population involved in transmission by 35% (95% CI 27 to 50%) is also predicted to reduce the *R*_*0*_^*tot,Sb*^ to beneath the threshold of 1 (Fig. 4*B*).

The impact of annual treatment of cattle was further explored in the longitudinal simulations described in *SI Appendix*, Fig. S9.

### Evidence for Density-Dependent Reduction in Schistosome Fecundity.

The observed worm count and miracidia per gram of feces data from 17 cattle infected with *S. bovis* at postmortem supported the hypothesis for a negative relationship between per-pair fecundity and increasing worm count (Spearman rank: *P* = 0.001, rho = −0.73) or increasing worm pair count (Spearman rank: *P* < 0.001, rho = −0.75). Data for small ruminants infected with *S. bovis* were sparse, but when small ruminants infected with all genotypes were included (*n* = 10; individuals infected with *S. bovis*, *S. curassoni*, and *S. curassoni* × *S. bovis* hybrids including coinfected individuals), correlation testing indicated weak evidence supporting negative density-dependent effects due to increasing worm count (Spearman rank: *P* = 0.08, rho = −0.59) and increasing pair count (Spearman rank: *P* = 0.056, rho = −0.62). Estimation of the parameters which described the density-dependent relationship for each species are described in detail in *SI Appendix*.

## Discussion

Against a background of global trends such as climate change, changing agricultural practices, and migration (2), all predicted to exacerbate the potential for both animal and human populations to encounter new infections, it is becoming increasingly important to consider all aspects of complex, multifaceted disease systems. Understanding the pathways and drivers of zoonotic spillover, the factors that may enable zoonotic diseases to spread within human populations, and the potential consequences (including hybridizations and introgressions) of interactions between multiple species and strains of pathogens is key to mitigating their impact (35).

Domestic animals often play a key role in cross-species transmission of zoonoses due to their close association with human populations (36, 37), and zoonoses affecting livestock represent a double burden to poor rural communities for whom livestock are a crucial source of income and economic security (38). Understanding the disease dynamics within zoonotic reservoirs is therefore also essential for prioritizing surveillance and predicting disease emergence (36).

While the prevalence of introgressed *S. haematobium* × *S. bovis* hybrids in the human population studied here was high, the burden of zoonotically acquired *S. bovis* was estimated to be very low, representing less than 0.2% of the total Haematobium group schistosome human worm burden. This implies that transmission of *S. bovis* from the livestock population to humans happens very infrequently in comparison to transmission of other genotypes. In this setting, the low level of *S. bovis* in the human population cannot be easily explained by spatial or behavioral barriers given that this is an area with high levels of cocirculating *S. bovis* and *S. haematobium*, where humans and livestock commonly share water access points. Prevalence of *S. bovis* in the cattle population in particular is very high and infected snails shedding both *S. bovis* and *S. haematobium* were commonly collected from the same water access points (19). This would indicate that a combination of *S. bovis* biology and human physiological factors are limiting the incidence of spillover rather than lack of exposure.

Given that the basic reproduction number for introgressed hybrids between *S. haematobium* and *S. bovis*, denoted *Hyb* within this framework, was estimated to be greater than one (robust to uncertainty analysis), this indicates that this genotype can be maintained within the human population in the absence of zoonotic spillover. Indeed, the estimated value for *R*_*0*_^*H,Hyb*^ is comparable to the estimated value for *S. haematobium* within this framework and other published estimates of the *R*_*0*_ for *S. haematobium* (39, 40). Classification of hybrids within the conceptual frameworks proposed for multihost systems (27, 28) is not intuitive, with formation of hybrids dependent on presence of *S. haematobium*. The results presented here indicate that while *S. bovis* can be considered a spillover parasite with a livestock reservoir, humans can be classified as maintenance hosts for *S. haematobium* × *S. bovis* hybrids, and that if introduced to a new population transmission could persist in the absence of a zoonotic reservoir.

The inference of the findings presented is that even if zoonotic spillover and hybridization in human hosts may occur infrequently in areas where *S. haematobium* and *S. bovis* are coendemic, hybrids will have the capacity to persist if introduced to new geographic localities in the presence of a suitable intermediate snail host. These findings are consistent with recent genomic studies on human patients from both Niger and Corsica which found evidence of *S. haematobium* × *S. bovis* hybrids in humans arising from historical hybridization events, estimated to have occurred up to 108 to 613 generations ago (41, 42). Furthermore, a recent phylogenetic analysis comparing *S. bovis* samples from cattle and miracidia from human samples in Senegal found no evidence of zoonotic spillover (43). This lends weight to the generalizability of the conclusion that hybridization between *S. bovis* and *S. haematobium* in human hosts is likely to be geographically isolated and that hybrid genotypes can persist in humans without contemporaneous zoonotic spillover.

Nevertheless, even low levels of zoonotic transmission are likely to become more epidemiologically significant in the context of elimination. Our model simulations predict that under conditions of increased MDA coverage, assuming stable rates of zoonotic transmission, hybrids will come to represent an increasing proportion of a diminished worm burden in the human population. This trend is observed despite assuming no increased fitness traits in the hybrid population, and assuming that praziquantel is equally efficacious against all worm genotypes. Key to this projected shift in parasite community composition is the presence of a livestock population which remains untreated, meaning the *S. bovis* reservoir will remain stable, with zoonotic transmission therefore coming to represent an increasing proportion of the overall force of infection on the human population.

The wider public health concerns for a growing predominance of schistosome hybrids include the observed potential for hybrid genotypes to have an increased host range (both definitive and intermediate), increased infectivity, and altered pathology (20, 22, 44, 45). For example, laboratory experiments have indicated that hybrids between *S. haematobium* and other schistosome species (*Schistosoma guineensis* and *S. matthei*) may exhibit increased infectivity to snail intermediate hosts and greater fecundity (46, 47). Increased fecundity, as well as being advantageous, may also be associated with increased morbidity in the human population, as the majority of pathology within definitive hosts is caused by inflammation associated with host immune response to eggs that become trapped in host tissues (12).

However, as such traits have not yet been demonstrated for hybrids between *S. haematobium* and *S. bovis*, our model assumes no increased infectivity to snails or fecundity to hybrids, nor a greater male sex ratio bias, which has also been demonstrated to confer advantage in interactions between different schistosomes (48–50). Further work may seek to clarify whether these traits are present in *S. haematobium* × *S. bovis* hybrids, but if such advantageous properties were present the predominance of these hybrids under increasing evolutionary pressure would be predicted to be even more rapid. Furthermore, traits such as a wider host range or increased morbidity would mean the projected shift in parasite community composition could have broader public health consequences in resurgence and reemergence scenarios, for example following cessation of MDA before local elimination is achieved, as an “escaped” worm population will be composed of a greater proportion of hybrids.

Our results clearly identify cattle as maintenance hosts for *S. bovis* in this region, as well as essential hosts, meaning transmission could not be maintained in their absence. In China, as prevalence of *Schistosoma japonicum* in humans became very low following sustained multifaceted control campaigns, the role of cattle as a zoonotic reservoir in certain regions became a critical barrier to elimination, and in many cases domestic bovines were removed (51, 52). The economic and cultural significance of cattle to communities in rural Senegal means removal of bovines would not be an acceptable solution here, and currently the only treatment option for livestock schistosomiasis is praziquantel chemotherapy. There are, however, valid concerns that widespread use of praziquantel in animal species may lead to development of resistance traits in the parasite population and that via zoonotic spillover and hybridization such traits could potentially be transmitted to parasites in human populations. Our study also provides evidence for density-dependent reduction in fecundity in naturally occurring *S. bovis* infections of cattle, with the presence of such density-dependent effects predicted to be a key factor in the spread of anthelmintic resistance as well as the observed resilience of helminths to control efforts despite relatively low estimates of *R*_*0*_ (53, 54). Furthermore, it has been hypothesized that untreated livestock populations may act as a *refugia,* ultimately slowing development of resistance (55, 56), highlighting the need to fully understand the nature of multihost transmission dynamics and appraise all possible outcomes when considering interventions targeting nonhuman populations.

Given the observed capability of hybrid schistosomes to infect a wider range of snail host species as compared to their parental schistosome species (20, 46, 47), the identification in this study of humans as maintenance hosts for *S. haematobium* × *S. bovis* hybrids highlights the potential for hybridization to exacerbate the emergence of schistosomiasis in new geographic locations, a concern exemplified by the recent outbreak in Corsica (25). In Zanzibar, one of the few areas in Africa currently targeted for interruption of transmission by the World Health Organization, *S. bovis* has recently been isolated for the first time from snail hosts (57). The introduction of *S. bovis* is thought to be associated with an expansion of cattle farming in Zanzibar and increased cattle imports following eradication of tsetse flies (57, 58), demonstrating the risk that changing agricultural practices and animal movement, in addition to human migration, may represent for emergence of schistosomiasis to new localities.

Much emphasis is now being placed on how rapid anthropogenic changes are increasing interactions between host species, thus enhancing opportunities for disease spillover and host adaptation. However, little attention has been given to how spillover can in turn lead to complex interactions between pathogen strains or species. Our study demonstrates that such interactions could play an important role in complex disease systems, such as that represented by Haematobium group schistosomiasis, and how elimination efforts against such diseases are not only challenged by, but themselves may influence, the impact of cross-species transmission dynamics. This demonstrates the importance of considering disease control from a multipathogen as well as multihost perspective, with the transdisciplinary approaches described here becoming more broadly relevant than ever in a rapidly changing world. As humanity increasingly faces novel challenges in the form of emerging and reemerging infections, understanding all aspects of the ecological processes that enable and drive the persistence and spread of threats to public health, both old and new, will be pivotal to alleviating their impact and is more critical now than ever.

## Materials and Methods

### Datasets and Data Collection.

The datasets used for to parameterize the model are taken from the surveys carried out in northern Senegal between 2015 and 2018; study sites, data collection, sample processing, and molecular analysis are described in detail in ref. 19.

Parasitological and epidemiological data were collected from human populations of the Senegal River Basin area (specifically nine villages around the Lac de Guiers and three periurban communities in the nearby town of Richard Toll), the livestock (cattle, sheep, and goats) which belong to these communities, and the sympatric snail populations. In this area, transmission sites (snail intermediate host habitats) are permanent water sources, meaning transmission is perennial, with water access points commonly shared by communities and their livestock. The Richard Toll/Lac de Guiers area was identified as having both a high prevalence of *S. haematobium* × *S. bovis* hybrid schistosomes in the human population and a high prevalence of *S. bovis* in the livestock population (19).

Briefly, schistosome egg counts per 10 mL of urine were estimated for human participants (taken as the mean from duplicate urine filtrations) and miracidia per gram of feces was estimated for livestock, based on microscopy of urine filters and miracidia hatching technique (MHT), respectively (miracidia being the first larval schistosome stage which infects the intermediate freshwater snail host). Duplicate Kato–Katz (KK) fecal slides from all livestock participants were also examined for the presence of eggs. Snail surveys were carried out at water access points used by people and animals in each community, and snails were individually exposed to light to enable shedding of cercariae, the second schistosome larval stage which infects mammalian definitive hosts (human and nonhuman) via skin penetration on contact with water. Miracidia and cercariae (from positive mammalian and snail hosts, respectively) were individually pipetted onto Whatman FTA cards (GE Healthcare Life Sciences) for DNA storage and molecular analysis.

Further livestock surveys were carried out in the abattoir of Richard Toll between November 2015 and April 2018 (cattle, sheep, and goats routinely slaughtered as part of the normal daily work of the abattoir), also detailed in ref. 19. On postmortem, mesenteric blood vessels were visually inspected for the presence of adult worms and all worms identified were removed via dissection. Dissecting microscopes were used to separate pairs shortly after collection to enable quantification of total pairs, total males, and total females for each positive animal before storing worms in RNAlater (Qiagen) for subsequent molecular analysis. Rectal feces were obtained, and two KK slides examined for the presence of eggs in addition to MHT, with miracidia individually counted from each fecal sample.

### Genotype Categorization of Worms, Miracidia, and Cercariae.

Methods for molecular analysis of worms, miracidia, and cercariae are described in detail in ref. 19. Briefly, individual *Schistosoma* DNA extracts were characterized by amplification of a partial fragment of the mitochondrial cytochrome *c* oxidase subunit 1 (*cox1*) and the complete nuclear ribosomal DNA internal transcribed spacer (ITS). The categorization of miracidia from definitive hosts (humans, cattle, sheep, and goats) described in ref. 19 was expanded upon to distinguish miracidia genotypes based on the worm pairs that are most likely to have given rise to the observed *cox1* and ITS signals, with chromatogram peak height used to distinguish likely F1 hybrids from early backcrosses. The miracidia classification used here comprises *S. haematobium* (*Sh*), *S. bovis* (*Sb*), and five genotype categories of hybrids/introgressions of *S. haematobium* with *S. bovis.* This system of classification is described in *SI Appendix*, Table S1. Categorization of miracidia in this way corresponds to seven different worm pair combinations in the human population of five distinct worm genotypes, indicated in Table 1 and Fig. 1.

The miracidia categorized as Hybrid2 and Hybrid3 represent both first generation (F1) and recently backcrossed hybrids. These include the products of initial cross-species pairings between *S. haematobium* and *S. bovis* (female *S. bovis* and male *S. haematobium* for Hybrid2 F1, and female *S. haematobium* and male *S. bovis* for Hybrid3 F1). Corresponding F1 worms arising from these miracidia were categorized as F1a and F1b Hybrid worms for Hybrid2 and Hybrid3, respectively, then miracidia produced by backcrosses between these F1a and F1b worms and *S. haematobium* were categorized as Hybrid2 Backcross and Hybrid3 Backcross. The Hybrid2 and Hybrid3 miracidia were subcategorized as F1 or F2/“Backcross” based on chromatogram peak heights. The worm genotype denoted *Hyb* then represents all hybrids that arise from any subsequent later generation pairings and backcrosses, and Hybrid1 miracidia represents all miracidia that are the product of pairings involving these *Hyb* worms, including backcrossing of these hybrids with *S. haematobium.* Note that miracidia that are the product of female *Hyb* with *S. haematobium* or female *Hyb* with male *Hyb* cannot be distinguished with current molecular methods. As no *S. bovis* miracidia were isolated from human hosts in this study (or any other studies in Senegal) it was assumed here that only livestock hosts can shed this species and *S. bovis* with *S. bovis* pairings in human hosts were not included in this framework.

As cercariae are produced by clonal expansion following infection of the snail intermediate host by a miracidium, cercariae genotype followed the same categorization as miracidia.

### Dynamic Transmission Model.

The mathematical transmission model presented here is a deterministic mean worm burden model, developed following the original work of May (59), further elaborated by Anderson and May (32) and May and Woolhouse (60). Aspects of the model have also been informed by the work of Morand et al. (49) in modeling another hybrid schistosome system (*S. haematobium* and *S. guineensis* in Cameroon).

A schematic representing the dynamic transmission model is given in Fig. 1 and parameter definitions, estimations, and sources of estimation together with definitions of model variables are given in *SI Appendix*, Table S2. The model is described briefly here, with full details given in *SI Appendix*.

Abundance and distribution of each worm genotype in each host populations is described by a negative binomial distribution ∼*NB*(*m*_*d,j*_*,κ*_*d*_), where *m*_*d,j*_ is the mean number of worms of genotype *j* in definitive host *d,* and where *κ*_*d*_ describes the degree of dispersion of worms in the host population, with a small *κ*_*d*_ representing increasing overdispersion and *κ*_*d*_ →∞ moving toward a Poisson distribution corresponding to worms randomly distributed within the population. All worm genotypes within a given host population were assumed to have the same degree of dispersion across host individuals, described by a single *κ*_*d*_ value.

The dynamics of mean worm burden in each definitive host population with respect to time are then given by the equation

$$\frac{dm_{d,j}}{dt}=A_{d,j}L_{j}-m_{d,j}\sigma_{d\text{.}}$$ [1]

In this model, the miracidia, snail, and cercariae stages of the schistosomiasis life cycle are collapsed and represented by a pool of free-living larval infectious material, with *L*_*tot*_ comprising all genotypes involved in the transmission cycle. The term *L*_*j*_ represents the component of this pool that on successful infection of a definitive host will produce worms of genotype *j*, and *A*_*d,j*_ represents a general daily rate of transmission from the infective stages *L*_*j*_ to host population *d*. Worm death rate in host population *d* is represented by $\sigma_{d}$. In humans, this is the sum of worm death rate in the absence of treatment (a function of the mean life expectancy of worms; years^−1^) and annual worm death rate due to treatment. Given that animals are not routinely treated for schistosomiasis in the study region, and the relatively short life expectancy of the livestock species included in this model, $\sigma_{d}$ in livestock is assumed to be the sum of worm death rate due to natural mortality of worms and worm death rate due to host mortality (61).

The dynamics of the environmental pool of infectious material are then described by the equation

$$\frac{dL_{j}}{dt}=\left[\sum_{d=H,C,S,G},\beta_{d,j},\psi_{d,j},\lambda_{d,j},{\bar{f}}_{d},N_{d}\right]-\gamma L_{j}.$$ [2]

Here $\beta_{d,j}$ represents a general transmission parameter from eggs/miracidia produced by worm pairs in host species *d* to the larval pool, where $\psi_{d,\;j}$ represents the mean number of all worm pair combinations per host in population *d* that contribute to larval stage *L*_*j*_ (see *SI Appendix*, Table S1 and *SI Appendix*). Mean daily egg shedding by a worm pair in host *d* is then given by $\lambda_{d,j}$ and the mean per-pair density-dependent reduction in fecundity in species *d* is given by ${\bar{f}}_{d}$. The population of host *d* is given by $N_{d}$ and decay rate of infectious stages in the environment is given by γ, which is assumed to be constant for all genotypes.

Transmission from the *S. bovis* component of the larval pool (*L*_*Sb*_) to the human population represents zoonotic spillover in this framework. Subsequently F1a and F1b hybrid miracidia (and worms which ultimately arise from these miracidia) are created from pairing of *S. bovis* with *S. haematobium* within the human host. When these F1 worms then pair with *S. haematobium* worms, eggs produced from these pairings create F1a and F1b Backcross miracidia. The backcrossed miracidia then contribute to the component of a general hybrid pool *L*_*Hyb*_ (which generates all later-generation hybrid worms, denoted *Hyb*), representing successful onward transmission of *S. bovis* genetic material here.

Following previous studies and existing models of schistosomiasis in humans (32, 62), and exploration of four possible models of the relationship between fecal egg shedding and *S. bovis* worm burden in in different livestock hosts (see *SI Appendix*, *Additional Results* and Table S5), a per-couple density-dependent reduction in fecundity is assumed to be described here by a negative exponential function (with declining fecundity in proportion to increasing worm burden of all genotypes).

The worm mating probability function used in this model follows that described by May and Woolhouse (60), and its use, including assumptions regarding mating interactions, is described in *SI Appendix*.

### Estimation of Mean Worm Burden, Dispersion, and Transmission Parameters.

Results from the KK and MHT tests together with the miracidia per gram count from the live animal survey detailed in ref. 19 were applied to a Bayesian framework to estimate mean worm burden ($m_{d,Sb}$) and dispersion parameters (*κ*_*d*_) for livestock populations. This framework was further developed to incorporate egg counts and genotyped data, in order to estimate the worm burden in each individual in the human study at baseline and the mean worm burden of each genotype (*m*_*H,j*_) together with the dispersion parameter *κ*_*H*_ for the human population.

Dynamic model transmission parameters $A_{d,j}$ and $\beta_{d,j}$ were estimated by assuming that transmission dynamics were at equilibrium at the time of data collection. For the livestock component of the model, this assumption is supported by the fact that *S. bovis* has been reported in the Senegal River Basin since the 1960s (63, 64), with no interventions systematically targeting the animal population or any systematic mollusciciding in this area. For the human component of the model, this assumption is also reasonable given that current control interventions targeting the human population, comprising MDA of praziquantel to school-aged children, have been in place since 2006/2007, and no significant difference was observed in the schistosomiasis prevalence or proportion of children shedding different schistosome genotypes between the 2016 and 2017 to 2018 surveys described in ref. 19.

In order to validate the worm burden estimates from the Bayesian analysis and assumptions regarding mating order, the expected percentages of total couples in the human population that would be composed of each pair combination (these proportions can be considered as the expected proportion of miracidia output of each category from humans) were compared to the observed proportions of miracidia and cercariae of each genotype (see *SI Appendix*, Table S4 and Fig. S5). The observed miracidia genotype percentages fall within the 95% CIs for the corresponding worm-pair estimates generated by the Bayesian output.

Further details of the Bayesian frameworks used, their implementation, and estimation of dynamic transmission parameters are given in *SI Appendix*.

### Estimation of *R*_*0*_ and Partitioning of *R*_*0*_ for the Multihost *S. bovis* System.

For macroparasites such as schistosomes, *R*_*0*_ is defined by Anderson and May (40) as the average number of mated adult female offspring produced during the lifespan of one mated female schistosome in the absence of constraints on population growth. If the value of *R*_*0*_ is less than one, i.e., the average female worm does not replace herself in the next generation, the parasite cannot persist. Formulation of the *R*_*0*_ for each genotype *j* within each host species *d* (*R*_*0*_^*d,j*^) within this system is described in *SI Appendix*.

Estimation of the *R*_*0*_ for the Hyb genotype in the human population (denoted *R*_*0*_^*H,Hyb*^) was used to evaluate the capacity of *S. haematobium* × *S. bovis* hybrids to be maintained in the human population in the absence of zoonotic spillover. If the average “*Hyb*” female does not replace herself in the next generation (*R*_*0*_ < 1) this genotype category could not be maintained without the creation of novel hybrids via zoonotic spillover.

The formulation for a single host species *R*_*0*_^*d,j*^ was extended to the multihost *S. bovis* system to calculate the overall *R*_*0*_ across the entire host species community, denoted *R*_*0*_^*tot,Sb*^. The next-generation matrix approach described by Roberts and Heesterbeek (65) was applied, where the “next generation” here refers to the transmission of infection from a definitive host to the environmental pool of infectious material and subsequent successful infection of another definitive host and generation of a new female worm. In a system with *n* definitive host species, the next-generation matrix (*K*) would be constructed as an *n* × *n* matrix, with each element *k*_*d,g*_ representing the expected number of female worms in definitive host species *d* that would be generated during the lifetime of a mated female worm in host population *g.* The diagonal elements of the matrix (where *d* = *g*) are therefore the within-species *R*_*0*_^*d,Sb*^.

The formulation of the next-generation matrix is given in *SI Appendix*. It assumes no spatial structuring of transmission, which was justified by the observation that households keeping cattle, sheep, and goats were found in all of the study villages and that these species generally share common water access points. The *R*_*0*_^*tot,Sb*^ was then calculated as the dominant eigenvalue of the transmission matrix *K.*

### Longitudinal Simulations: Predicting the Impact of Eliminating Zoonotic Transmission and the Impact of Interventions Targeting the Animal Population.

Longitudinal simulations of the dynamic model were implemented to examine the potential impact of removing zoonotic transmission under current levels of treatment coverage in the human population and also under enhanced treatment coverage levels. The impact on the burden of each worm genotype (and total worm burden) was simulated under MDA treatment coverage levels of 50% (assumed baseline) and increased coverage levels of 75% and 90%, both with current assumed levels of zoonotic transmission (*A*_*H,Sb*_ estimated from Bayesian output) and in scenarios where there is no zoonotic transmission (simulated by setting *A*_*H,Sb*_ to zero). The proportion of the total mean worm burden that is composed of hybrids was calculated within each simulation. For all scenarios simulated here it is assumed that *S. bovis* continues to circulate within the animal population at equilibrium, with *L*_*Sb*_ therefore assumed to be stable.

Cattle were identified as both essential and maintenance hosts for *S. bovis* transmission (*Results*), and therefore the impact on *R*^*tot,Sb*^ by annually treating cattle with praziquantel was explored, simulating coverage rates of 0 to 100%. Given concerns regarding the practicalities and potential negative consequences of treating livestock with praziquantel (56) the impact on *R*_*0*_^*tot,Sb*^ of removing 0 to 100% of the cattle population from schistosomiasis transmission was also evaluated. This was simulated by reducing *N*_*C*_ by a factor of 0 to 100%.

Longitudinal dynamic model simulations were also carried out to assess the impact of annually treating the cattle population on the mean worm burden of each livestock population and on the density of infectious *S. bovis* material in the environment, which has been identified as the source of zoonotically acquired *S. bovis* infections in humans (additional results in *SI Appendix*).

### Model Implementation and Uncertainty Analysis.

Due to the uncertainty around many of the parameter estimates for the livestock component of the model, which were derived from the data, literature, and government resources, these were assigned a distribution of plausible values for each host species for the estimation of transmission rates and *R*_*0*_^*d,j*^. A random set of values for the population estimates (*N*_*d*_), death rate of worms ($\sigma_{d}$), decay rate of infectious material in the environment (*γ*), and daily mass of fecal production for each species (*g*_*d*_) were generated using the Latin hypercube sampling method (LHS) to efficiently sample the parameter space (*80,81*), drawing from distributions given in *SI Appendix*, Table S2 to generate 1,000 sets of parameters.

The LHS method was not suitable for exploring the parameter space for the values for mean worm burden, worm dispersion, fecundity, and density dependence ($m_{d,Sb}$, *κ*_*d,*_ *a*_*d,Sb*_, and *b*_*d*_) as these were jointly estimated within the Bayesian framework described (the LHS method, by selecting independent values for each variable, would not account for the covariance between parameter estimates). Therefore, to account for the uncertainty in these parameter estimates, 1,000 iterations containing estimates for these four parameters were randomly selected from one posterior Markov chain Monte Carlo (MCMC) chain (following discarding of burn-in period) for each of the livestock host species. Thus, 1,000 sets of all parameters were created and used to generate 1,000 values of $\beta_{All,Sb},\;\lambda_{d,Sb}$, *A*_*d,Sb*_, *R*_*0*_^*d,Sb*^, and *R*_*0*_^*tot,Sb*^ and thereby calculate the median and 95% CIs for these estimates (where 95% CIs here refer to the 95-centile range).

In order to account for the uncertainty in the Bayesian estimation for key parameters in the human population (the dispersion parameter, *κ*_*H*_, and mean worm burdens for each genotype in the human population $m_{H,j}$), one posterior MCMC chain was randomly selected (5,000 iterations after discarding of burn-in period and thinning) and the transmission parameters $A_{H,j}$and $B_{H}$ were calculated for each iteration. These 5,000 estimations of each transmission parameter were then used to generate 5,000 estimations of *R*_*0*_^*H,Hyb*^ and *R*_*0*_^*H,Sh*^. Similarly, for each iteration the estimated worm burdens were used to estimate expected mean couples of each pairing combination and the proportion of total couples that would be composed of each pair combination. Median values are presented and 95th centiles given as 95% CIs.

For all longitudinal simulations 1,000 sets of parameter values were applied to create 1,000 simulations. These were generated as described above for parameters relating to the livestock population. For parameters relating to the human population, 1,000 iterations were randomly chosen from one posterior MCMC chain from the Bayesian analysis output, with transmission parameters estimated for each.

### Ethical Approval.

Ethical approval was provided by 1) Imperial College London application 03.36, 2) the Clinical Research Ethical Review Board at the Royal Veterinary College (London) application URN20151327, and 3) the Comité National d’Ethique pour la Recherche en Santé (Dakar, Senegal) application SEN15/68. Written informed consent was obtained from all adult participants, children’s parents or guardians, and livestock owners. Infected adults and children were treated with 40 mg/kg of praziquantel. Livestock owners were offered standard anthelmintics for infected animals.

## Supplementary Material

Supplementary File

## Data Availability

Molecular sequences have been deposited in GenBank (accession nos. MT580946–MT580963, MT579420–MT579449, MN593376, MN593380, MN593384, MN593388, MN593392, MN593396, MN593400, and MN593404). Code for dynamic models and data files to run dynamic model code, together with statistical model code, are available at https://github.com/AnnaMB123/Hybrid_schisto_PNAS. Molecular samples are available through the Schistosomiasis Collection at the Natural History Museum (SCAN; https://scan.myspecies.info).
