# Supplementary material for: Spillover, hybridization, and persistence in schistosome transmission dynamics at the human–animal interface
Source: Proc Natl Acad Sci U S A. 2021 Oct 6;118(41):e2110711118. doi: 10.1073/pnas.2110711118 (PMC8521685; doi:10.1073/pnas.2110711118)
Supplement: Supplementary File [file pnas.2110711118.sapp.pdf]

PNAS
www.pnas.org

Supplementary Information for

Spillover, hybridization, and persistence in schistosome transmission dynamics at the human-animal interface

Authors: Anna Borlase, James W. Rudge, Elsa Léger, Nicolas D. Diouf, Cheikh B. Fall, Samba D. Diop, Stefano Catalano, Mariama Sene & Joanne P. Webster

Corresponding author: Anna Borlase

Email: anna.borlase@bdi.ox.ac.uk;

This PDF file includes:

Supplementary Text

Figures S1 to S10

Tables S1 to S6

References

Supplementary Text: Extended Materials and Methods

Datasets and data collection

The datasets used for to parameterize the model are taken from the surveys carried out in Northern Senegal between 2015 and 2018; study sites, data collection, sample processing and molecular analysis are described in detail in (*1*).

This work focuses on populations of the Senegal River Basin area, specifically the human, livestock (cattle, sheep and goat) and snail populations of nine villages around the Lac de Guiers and three peri-urban communities in the nearby town of Richard Toll. This area was identified as having both a high prevalence of *Schistosoma haematobium* x *Schistosoma bovis* hybrid schistosomes in the human population, and a high prevalence of *Schistosoma bovis* in the livestock population (*1*).

Briefly, during surveys of the human population carried out between 2016 and 2018 schistosome egg counts per 10ml of urine were taken as the mean from duplicate 10ml urine filtrations examined microscopically. Urine filters which were positive for schistosome eggs were placed in fresh water to facilitate hatching of miracidia- the first larval schistosome stage which infects the intermediate freshwater snail host, and miracidia were individually pipetted onto Whatman FTA cards (GE Healthcare Life Sciences, UK) for DNA storage and molecular analysis.

During surveys of sympatric cattle, sheep and goats belonging to the communities in the study, faeces samples were obtained via rectal sampling and duplicate Kato-Katz (KK) slides prepared for each sample (*2*) followed by examination for the presence of eggs. A random subset of faeces samples were also processed following the miracidia hatching technique (MHT) described in (*1*). This enabled collection and quantification of miracidia from each sample. Snail surveys were carried out at water access points used by people and animals in each community, and snails individually exposed to light to enable shedding of cercariae- the second schistosome larval stage which infects mammalian definitive hosts (human and non-human) via skin penetration on contact with water.

Further livestock surveys were carried out in the abattoir of Richard Toll between November 2015 and April 2018 (cattle, sheep and goats routinely slaughtered as part of the normal daily work of the abattoir), also detailed in (*1*). On post-mortem, mesenteric blood vessels were visually inspected for the presence of adult worms and all worms identified were removed via

dissection. Dissecting microscopes were used to separate pairs shortly after collection to enable quantification of total pairs, total males and total females for each positive animal, before storing worms in RNA-later (QIAGEN, Hilden, Germany) for subsequent molecular analysis. Rectal faeces were obtained, and two KK slides examined for the presence of eggs in addition to miracidia hatching, with miracidia individually counted from each faeces sample.

Free-swimming miracidia from all positive livestock samples, and cercariae from infected snails were individually stored on Whatman FTA Classic cards for DNA storage and molecular analysis.

Genotype categorization of worms, miracidia and cercariae

Methods for molecular analysis of worms, miracidia and cercariae are described in detail in (*1*). Briefly, individual *Schistosoma* DNA extracts were characterised by amplification of a partial fragment of the mitochondrial cytochrome c oxidase subunit 1 (*cox1*) and the complete nuclear ribosomal DNA internal transcribed spacer (ITS). The categorization of miracidia from definitive hosts (humans, cattle, sheep and goats) described in (*1*) was expanded upon to distinguish miracidia genotypes based on the worm pairs that are most likely to have given rise to the observed cox1 and ITS signals, with chromatogram peak height used to distinguish likely F1 hybrids from early backcrosses. The miracidia classification used here comprises *S. haematobium* (*Sh*), *S. bovis* (*Sb*) and five genotype categories of hybrids/introgressions of *S. haematobium* with *S. bovis.* This system of classification is described in Table S1. Categorization of miracidia in this way corresponds to seven different worm pair combinations in the human population of five distinct worm genotypes, indicated in Table 1 and Figure 1 in the main text.

The miracidia categorised as Hybrid2 and Hybrid3 represent both first generation (F1) and recently backcrossed hybrids. These include the products of initial cross-species pairings between *S. haematobium* and *S. bovis* (female *S. bovis* and male *S. haematobium* for Hybrid2 F1, and female *S. haematobium* and male *S. bovis* for Hybrid3 F1). Corresponding F1 worms arising from these miracidia were categorised as F1a and F1b Hybrid worms for Hybrid2 and Hybrid3 respectively, then miracidia produced by backcrosses between these F1a and F1b worms and *S. haematobium* were then categorised as Hybrid2 Backcross and Hybrid3 Backcross. The Hybrid2 and Hybrid3 miracidia were sub-categorised as F1 or F2/"Backcross" based on chromatogram peak heights. The worm genotype denoted *Hyb* then represents all hybrids that arise from any subsequent later generation pairings and backcrosses, and Hybrid1

miracidia represents all miracidia that are the product of pairings involving these *Hyb* worms, including backcrossing of these hybrids with *S. haematobium.* Note that miracidia that are the product of female *Hyb* with *S. haematobium* or female *Hyb* with male *Hyb* cannot be distinguished with current molecular methods. As no *S. bovis* miracidia were isolated from human hosts in this study (or any other studies in Senegal) it was assumed here that only livestock hosts can shed this species and *S. bovis* with *S. bovis* pairing in human hosts were not included in this framework.

As cercariae are produced by clonal expansion following infection of the snail intermediate host by a miracidium, cercariae genotype followed the same categorization as miracidia.

Dynamic transmission model

The transmission model presented here is a deterministic mean worm burden model, developed following the original work of May (*3*), further elaborated by Anderson and May (*4*) and May and Woolhouse (*5*). Aspects of the model have also been informed by the work of Morand and colleagues (*6*) in modelling another hybrid schistosome system (*S. haematobium* and *Schistosoma guineensis* in Cameroon).

The model structure was based on the multi-host, multi-parasite transmission cycle for Haematobium group schistosomes proposed in (*1*), which was informed by molecular and epidemiological findings from surveys of human, snail and livestock populations. Key aspects of the system which are incorporated into the model include zoonotic transmission of *S. bovis* from the livestock reservoir to the human population, interactions between worm genotypes including formation of F1 *S. bovis* x *S. haematobium* hybrids in the human host via bi-directional cross-species pairings, and onward transmission of hybrid genotypes via backcrossing of F1 worms.

A simplified schematic of the dynamic transmission model is given in the main text, Figure 1. Parameter definitions, estimations, sources of estimation together with definition of model variables are given in Table S2.

In this multi-host framework the definitive hosts are humans (*H*), cattle (*C*), sheep (S) and goats (*G*), ($d \in \{H, C, S, G\}$). The dynamic variables in the model are the abundance of worms in the definitive host populations of the five schistosome worm genotypes designated in Table S1 ($j \in \{Sh, Sb, F1a, F1b, Hyb\}$).

Abundance and distribution of each worm genotype in each host populations is described by a negative binomial distribution $\sim NB(m_{d,j}, \kappa_d)$, where $m_{d,j}$ is the mean number of worms of genotype j in definitive host d, and where κ_d describes the degree of dispersion of worms in the host population, with a small κ_d representing increasing overdispersion and $\kappa \rightarrow \infty$ moving towards a Poisson distribution corresponding to worms randomly distributed within the population.

Dispersion of all worm genotypes is assumed to be the same in each host population and described by a single κ_d value.

The dynamics of mean worm burden in each definitive host population with respect to time are then given by the equation:

$$\frac{dm_{d,j}}{dt} = A_{d,j} L_j - m_{d,j}\, \sigma_d \qquad [1]$$

In this model, the miracidia, snail and cercariae stages of the schistosomiasis life cycle are collapsed and represented by a pool of free-living larval infectious material, with L_{tot} comprising all genotypes involved in the transmission cycle. The term L_j represents the component of this pool that on successful infection of a definitive host will produce worms of genotype j, and $A_{d,j}$ represents a general transmission rate from the infective stages L_j to host population d. Worm death rate in host population d is represented by σ_d. In humans, this is the sum of worm death rate in the absence of treatment (a function of the 4 year mean life expectancy of worms; years^{-1}) and annual worm death rate due to treatment. Annual worm death rate is given as the inverse of the product of praziquantel efficacy (95%), and annual MDA coverage (50% assumed at baseline; varied in longitudinal simulations (*7–9*)). Given that animals are not routinely treated for schistosomiasis in the study region, and the relatively short life expectancy of the livestock species included in this model, in livestock σ_d is assumed to be the sum of worm death rate due to natural mortality of worms (4 years^{-1}) and worm death rate due to host mortality (4-8 years^{-1} for cattle and 2-5 years^{-1} for small ruminants) (*9*).

The dynamics of the environmental pool of infectious material are then described by the equation:

$$\frac{dL_j}{dt} = \left[\sum_{d=H,C,S,G} \beta_{d,j} \psi_{d,j} \lambda_{d,j} \bar{f}_d N_d \right] - \gamma L_j \quad [2]$$

Here $\beta_{d,j}$ represents a general transmission parameter from eggs/miracidia produced by worm pairs in host species *d* to the larval pool, where $\psi_{d,j}$ represents the mean number of all worm pair combinations per host in population *d* that contribute to larval stage L_j (see Table S1). Mean daily egg shedding by a worm pair in host *d* is then given by $\lambda_{d,j}$ and the mean per-pair density-dependent reduction in fecundity in species *d* is given by $\bar{f}_d$. The population of host *d* is given by N_d and decay rate of infectious stages in the environment is given by γ, which is assumed to be constant for all genotypes.

Transmission from the *S. bovis* component of the larval pool (L_{Sb}) to the human population represents zoonotic spillover in this framework. Subsequently F1a and F1b hybrid miracidia and then worms are created from pairing of *S. bovis* with *S. haematobium* within the human host. When these F1 worms then pair with *S. haematobium* worms, eggs produced from these pairings create F1a and F1b Backcross miracidia. The backcrossed miracidia then contribute to the component of a general hybrid pool L_{Hyb} (which generates all later generation hybrid worms, denoted *Hyb*), representing successful onward transmission of *S. bovis* genetic material here.

Given that there is currently no evidence confirming variable egg shedding by worm pairs of different genotypes in this human population, the egg shedding parameter and density-dependent parameters are assumed to be the same for all genotypes, and denoted a_H and b_H. The formulation for the mean daily egg shedding by all worm pairs in a human host is given as:

$$\lambda_H = a_H u_H \quad [3]$$

Where a_H represents the mean number of eggs per 10ml produced per worm pair in the absence of density-dependent effects and u_H represents urine production by a human in the population (estimated as total daily urine production divided by 10 to keep a_H consistent with the 10ml samples in the data).

In livestock host population *d,* the mean daily egg shedding by a mated *S. bovis* female is then given by:

$$\lambda_H = a_{d,Sb} g_d \qquad [4]$$

Here g_d represents the mean daily faecal output of host *d* in grams and $a_{d,Sb}$ represents the per gram egg output per mated female in the absence of density-dependent constraints.

Following previous studies and existing models of schistosomiasis in humans (*4*, *10*), and exploration of four possible models of the relationship between faecal egg shedding and *S. bovis* worm burden in in different livestock hosts (see additional results table S5 page 44), a per-couple density-dependent reduction in fecundity is assumed to be described here by a negative exponential function (with declining fecundity in proportion to increasing worm burden of all genotypes). The mean per-pair density-dependent reduction in fecundity is then given by:

$$\overline{f_d} = \frac{\sum_{w_F=0}^{\infty} z^{\left(\frac{w_F}{q_d}-1\right)} * w_F * p\left(w_F\right)}{m_{d,tot} * q_d} \qquad [5]$$

Where b_d characterises the degree of the density-dependent effect:

$$z = e^{-b_d} \qquad [6]$$

The mean burden of female worms of all genotypes in the each host population is given by $m_{d,tot}$, and q_d is the proportion of all worms in the host that are female. The negative binomial probability $p(w_F,)$ that a host has w_F female worms is then given by:

$$p(w_F) = \frac{\left(w_F + \kappa_d - 1\right)!}{w_F!(\kappa_d - 1)!}\left(\frac{\kappa_d}{m_{d,tot}q_d + \kappa_d}\right)^{\kappa_d}\left(\frac{m_{d,tot}q_d}{m_{d,tot}q_d + \kappa_d}\right)^{w_F} \qquad [7]$$

With κ_d the dispersion parameter as described previously.

Worm mating probability and interactions: Livestock hosts

In livestock where the only schistosome pairing considered in this framework is between male and female *S. bovis* worms, the expression for the mean expected number of mated worm pairs in host *d* is given by:

$$\psi_{d,jF\_jM} = m_{d,j} q_{d,j} \phi_{d,jF\_jM}(q_{d,j}, m_{d,j}, \kappa_d) \qquad [8]$$

This is the product of the mean worm burden in each host species, $m_{d,j}$, the proportion of worms that are female ($q_{d,j}$), and the mating probability (ϕ_{d,jF_jM}). The mating probability is defined as the probability that a given female of genotype *j* in host *d* will be mated with a male of genotype *j,* assuming here there are no other genotypes in host population *d,* and assuming monogamous mating.

The formulation for the mating probability (ϕ_{d,jF_jM}) is given in equations 9-13. This mating function follows that described by May and Woolhouse (*5*) which allows for biased sex ratios. In each host population *d* this probability is a function of the mean worm burden ($m_{d,j}$) dispersion parameter (κ_d) and proportion of worms that are female ($q_{d,j}$).

$$\phi_{d,jF\_jM} = J(q_{d,j}) - 2(1-q_{d,j}) F(\kappa_d, \alpha_{d,j}, \omega_{d,j}) \qquad [9]$$

Where *J* is defined as:

$$J(q_{d,j}) = 1 \quad \text{for} \quad q_{d,j} \le 0.5$$

$$J(q_{d,j}) = (1-q_{d,j}) / q_{d,j} \quad \text{for} \quad q_{d,j} > 0.5 \qquad [10]$$

and *F* is the integral:

$$F(\kappa_d, \alpha_{d,j}, \omega_{d,j}) = \frac{(1-\alpha_{d,j})^{\kappa_d+1}}{\pi} \int_0^{\pi} \frac{\left[\sin^2\theta\right] d\theta}{[1+\alpha_{d,j}\omega_{d,j}\cos\theta]^{\kappa_d+1}[1+\omega_{d,j}\cos\theta]} \qquad [11]$$

and where $\alpha_{d,j}$ and $\omega_{d,j}$ are defined:

$$\alpha_{d,j} = m_{d,j} / (m_{d,j} + \kappa_d) \qquad [12]$$

$$\omega_{d,j} = 2((1 - q_{d,j})q_{d,j})^{0.5} \qquad [13]$$

Worm mating probability and interactions: human hosts

In the model framework used here, multiple worm genotypes may be present in human hosts. Equation 14 shows the formulation for the mean expected number of worm pairs of each combination where multiple genotypes may be present in host population *d.*

Here ψ_{d,jF_kM} represents the mean number of pairs comprising a female of worm genotype *j* and male of genotype *k* (*k* ∈ {*Sh, Sb, F1a, F1b, Hyb*}).

$$\psi_{d,jF\_kM} = m_{d,j(UF)}\phi_{d,jF\_kM}(q_{d,jF\_kM}, m_{d,j(UF)}, m_{d,j(UM)}, \kappa_d) \qquad [14]$$

Following Morand and Colleagues (*6*) and again assuming monogamous mating of worms, this is the product of the mean number of female available/unmated worms of genotype *j* in the host population, denoted ($m_{d,j(UF)}$), and the mating function ϕ_{d,jF_kM} which represents the probability that a female of genotype *j* is mated with a male of genotype *k.*

Due to the number of possible genotype mating combinations in the human hosts, in a mean worm burden framework and following Morand and colleagues (*6*) it was necessary to make some assumptions regarding the interactions and mating preferences of worms, and therefore the expected mean number of worms available for pairing at any time point. Due to the impossibility of observing worm interactions in the human population, assumptions were made which are parsimonious, but considered realistic, informed by observed data and the literature.

A male sex ratio bias in *S. bovis* within livestock was identified (see supplementary text), and a similar sex bias has been observed in experimental studies of other schistosome species (*11*). Within a mean worm burden framework with an assumed male sex bias and a worm population that is overdispersed, the mating probability for a given female will be nearly 100%. It is assumed here, following Morand and colleagues (*6*), that *S. haematobium* males will preferentially pair with *S. haematobium* females, given that the majority of observed miracidia were *S. haematobium* (see Table S4). However if it were to be assumed that *S. haematobium* males were dominant over all other genotypes, miracidia from alternative pairings that involve *S. haematobium* females (Hybrid3 F1 and Hybrid3 Backcross), would only be observed if there was relatively much larger burden of males of the genotypes involved in these pairings (specifically *S. bovis* and F1b), which only occasionally form pairs with the rarely available *S. haematobium* females, leaving the majority of *S. bovis* and F1b males unmated. It was considered more conservative to assume that the relative proportions of observed miracidia genotypes from an individual host will more closely reflect the proportions of their parental genotypes rather than each host potentially having a large unpaired worm burden. Furthermore, initial exploration of models assuming *S. haematobium* dominance over all genotypes exhibited problems regarding convergence. Therefore it was assumed that S. *bovis* and F1b genotype males, when present in a human host, would have a high probability of forming a pair with a *S. haematobium* female by applying a mating hierarchy in which these genotypes will pair with females preferentially over *S. haematobium* males.

The following order is therefore assumed regarding the seven possible mating combination in humans:

i) F1b males will first mate with *S. haematobium* females;
ii) Any *S. bovis* males will then mate with remaining *S. haematobium* females;
iii) *S. haematobium* males will then pair with *S. haematobium* females unmated by F1b or *S. bovis* males;
iv) *S. haematobium* males unpaired with *S. haematobium* females will then be available to pair with *Hyb* females;
v) *Hyb* males will pair with *Hyb* females unpaired with *S. haematobium* males;
vi) Any unmated *S. haematobium* males will then pair with F1a females;
vii) Any *S. haematobium* males will then pair with *S. bovis* females.

Within the dynamic model framework, the mean numbers of each pairing were calculated sequentially following these assumptions. Thus, for each combination the mean expected available worm burden for the pairing ($m_{d,j(UF)}$ and $m_{d,k(UM)}$ for females and males respectively) were calculated as the mean worm burden of each genotype/sex involved in the pairing, minus the mean number of pairs including this worm type that precede it in the mating order.

The mating probability is then formulated in the same way as that described in equations 9-13. Here $m_{d,j}$ will be substituted with the sum of the mean number of male and female worms available for the pairing ($m_{d,j(UF)}$ and $m_{d,k(UM)}$), the aggregation parameter κ_d will be as defined previously, and the proportion of the combined "available" worm population that is female will be given by:

$$q_{d,jF\_kM} = \frac{m_{d,j(UF)}}{m_{d,j(UF)} + m_{d,j(UM)}} \quad [15]$$

Selection of human datasets and imputation of genotype data

Due to the low number of adults in the human survey for whom molecular material was analysed (n=4 in 2016; n=14 in 2017-18), and the very low egg shedding from the adults sampled compared to the children (mean 12 eggs/10ml in adults, range 1-58; mean 56 eggs/10ml in children, range 1-990), the model is parameterised using data from the schools-based survey, which included children aged 5-17 years old.

For all individuals included in the surveys, mean eggs per 10ml of urine was recorded, and for those individuals where molecular material was available, a median of five miracidia were analysed (range 1-8) and assigned one of the six genotype categories described in S1. Molecular material and mean egg counts (per 10ml) were available from 165 (63%) of 264 positive children from the 2016 survey and from 154 (56%) of 275 positive children from the 2017-18 survey. There was no significant difference in the proportion of children positive in each survey (1 d.f, $\chi^2 = 0.031$, p=0.86), nor the proportion positive for each of the genotype categories (Fishers exact, p=0.57). Estimation of baseline worm burden counts was therefore based on the 2016 dataset due to the greater proportion of individuals where molecular data were available.

Molecular data were missing for 99 positive children from the 2016 survey, and it was therefore necessary to substitute this missing data using imputation. Imputation was carried out using multivariate imputation by chained equations (MICE) whereby plausible values for missing data are drawn from a distribution modelled specifically for each missing entry, implemented using the R package "mice" version 3.4.0 (*12*). Predictive mean matching (pmm) was used to create an imputed set of miracidia of designated genotype categories for each individual, using village, age and mean eggs per 10ml as predictor variables. The distribution of five imputed datasets was compared to the observed data to confirm validity of the imputation approach, and no significant differences were found between the imputed datasets and the original data (Fisher's exact 2-sided: Imputation 1 p=0.95; Imputation 2 p=0.98; Imputation 3 p=0.96; Imputation 4 p=0.79; Imputation 5 p=0.97). Imputation 2 then was randomly selected for use in further analysis and parameter estimation for the human components of the model.

Bayesian approach to characterizing relationship between worm count and egg count in naturally-occurring *S. bovis* infections of livestock

Given the lack of available data in the literature on the daily egg shedding by *S. bovis* in naturally infected livestock hosts, the relationship between worm burden and egg shedding rates was estimated using data from the abattoir surveys described in (*1*).

Total worm count estimates were available for 19 cattle, eight goats and two sheep positive for *S. bovis.* Small ruminant (sheep and goat) data were pooled due to the low number of worm positive individuals. Miracidia counts per gram of faeces following positive MHT were used as a proxy measure for eggs per gram of faeces.

Density-dependent reduction in worm fecundity as an individual's worm burden increases has been observed in many macroparasite populations, including schistosomes (*13–15*). This is often described by a negative exponential relationship, or alternatively a negative power relationship (*10*, *16*). Due to the imperfect nature of the MHT for estimation of egg count and the worm collection method, a Bayesian framework incorporating this uncertainty was used to estimate the per mated-female fecundity parameter, $a_{d,Sb}$, and a density-dependent parameter $b_{d},$ considering different possible formulations of the density-dependent effect on fecundity due to increasing worm burden.

Due to the non-normality of the worm count data (Shapiro Wilk test for normality $p<0.001$ for all species), worm count data and per-couple fecundity estimates were tested for negative correlation using Spearman's rank test for correlation, which would indicate a negative association between worm burden and fecundity.

Four models were considered for defining $f\left(w\right)$, the per-female density-dependent reduction in fecundity for an individual host with worm burden *w*. The first model assumes the density dependent effect is described by a power relationship with density-dependent reduction proportional to total worm burden:

$$f\left(w\right)=w^{\left(-b_d\right)} \qquad [16]$$

And a second model assumes density dependence is described by power relationship proportional to total burden of worm pairs (w_p):

$$f\left(w\right)=w_p^{\left(-b_d\right)} \qquad [17]$$

Similarly, a third and fourth model assumed density-dependence described by an exponential function proportional to either total burden of worms or total number of worm pairs in an individual:

$$f\left(w\right)=z^{\left(w-1\right)} \qquad [18]$$

$$f\left(w\right)=z^{\left(w_p-1\right)} \qquad [19]$$

Where:

$$z=e^{-b_d} \qquad [20]$$

Following the framework used for the dynamic model, the true worm burden of each individual in the dataset, *w[i],* is assumed to be drawn from a negative binomial distribution:

$$w[i] \sim NB(m_{d,Sb}, \kappa_d) \quad [21]$$

Given the imperfect nature of the worm collection method, the observed worm burden, *ow[i]*, is assumed to be a binomial sample of the true worm burden, where θ represents the mean worm detection rate (i.e., the probability that any given worm in a host is observed and counted during the post-mortem examination):

$$ow[i] \sim Binomial(\theta, w[i]) \quad [22]$$

A uniform prior distribution (Unif(0.9,1)) was assigned for the worm detection rate θ. The observed miracidia count (*om[i]*) was then assumed to be a sample from a Poisson distribution with mean given as the expected number of eggs (*ee[i])* in that individual:

$$om[i] \sim Poisson(ee[i]) \quad [23]$$

And the expected egg count was then given by:

$$ee[i] = w_p[i] * a_{d,Sb} * f[i] * g[i] \quad [24]$$

Where $w_p[i]$ is the total number of worm pairs in host *i*, *g[i]* is the weight of the faeces sample in grams, and *f[i]* is the density dependent reduction in fecundity, described by either the power function or exponential as given previously.

The parameter $a_{d,Sb}$ was assigned a wide, uninformative uniform prior distribution (Unif(0.01,100)). Having established evidence indicative of negative density-dependence in this dataset (see results main text) but in order to ensure parameter estimates were driven by the observed data, the parameter b_d was assigned a wide uniform prior distribution (Unif(-10,0)) for both exponential and power models of density-dependence. A value of b_d=0 would indicate no density-dependent effect and the lower limit of -10 extends well below ranges of negative density-dependent parameters used in similar models (*10*, *17*, *18*).

A schematic of the Bayesian model for parameter estimation from the abattoir data is given in Figure S1.

Bayesian approach to estimating mean worm burden and dispersion parameters for the livestock populations

The potential biases in the age, health status and geographical origin of the abattoir population meant abattoir data were not suitable for estimating the mean worm burden and dispersion in the live animal population for each definitive host species. Therefore the estimates for $a_{d,Sb}$ and b_d generated from the abattoir data were used to create priors which were utilised in a Bayesian framework (modified from that described above), represented schematically in Figure S2. Results from the KK and MHT tests together with the miracidia per gram count from the live animal survey detailed in (*1*) were applied to the framework to estimate $m_{d,Sb}$ and κ_d respectively.

The true worm burden of each individual was assumed to be drawn from the population-level negative binomial, with the distribution truncated at the maximum estimated worm burden in that species from the abattoir model to ensure estimates were biologically plausible. Weakly informative priors were used for the mean worm burden based on the observed worm counts in slaughtered animals, and weakly informative priors were used for the dispersion parameters (Table S6)

To ensure all individuals positive for either the KK or MHT test were always "assigned" at least one worm pair by the Bayesian model, the binary results (positive or negative) for the two diagnostic tests KK and MHT (applied in parallel to each individual), were modelled as a Bernoulli process. The probabilities were the product of the individual's "true" status as positive or negative for worm pairs (*Pair_pos[i]*) and the probability of observing each combination of results in that host species, based on the test sensitivities (any individual positive on either test must have had at least one worm pair), and the covariance between disease positive animals (covDp).

*p1[i] ~ Bernoulli ((SeKK*SeMHT+covDp)*Pair_pos[i])* [25]

p2[i] ~ Bernoulli ((SeKK(1-SeMHT)-covDp)*Pair_pos[i])* [26]

*p3[i] ~ Bernoulli(((1-SeKK)*SeMHT-covDp)*Pair_pos[i])* [27]

p4[i]= 1- (p1[i] + p2[i] +p3[i]) [28]

Where:

p1= (KK+,MHT+)

p2 = (KK+,MHT-)

p3 = (KK-,MHT+)

p4 = (KK-,MHT-)

This framework does not allow for false positives, assuming therefore specificity of both tests is 100%. This was considered to be a reasonable assumption given that the MHT and KK tests are based on microscopic visualisation of clearly identifiable schistosome eggs or miracidia.

Prior distributions for test sensitivities for the KK and MHT tests (SeKK and SeMHT respectively) were described using Beta distributions defined by parameters α and β ($\sim Beta(\alpha,\beta)$). The mean (μ) and standard deviation (σ) of test sensitivity estimates derived from abattoir data in (*1*) were used to generate values for α and β for use as priors in estimation of the live animal parameters, where:

$$\alpha = \mu\left(\frac{(1-\mu)\mu}{\sigma^2}\right) - 1 \qquad [29]$$

and:

$$\beta = (1-\mu)\left(\frac{(1-\mu)\mu}{\sigma^2} - 1\right) \qquad [30]$$

The test sensitivity priors assigned for the MHT and KK tests are given in Table S3. Median estimates of the *CovDp* for each species estimated in (*1*) was also used here (0.04 for cattle, 0.03 for small ruminants).

As in equations 23 and 24 above, the observed miracidia count for each sample was assumed to be a sample from a Poisson distribution with mean being expected egg count, which is a function of the number of worm pairs ($w_p[i]$), per pair fecundity ($a_{d,Sb}$), and density dependent reduction in fecundity ($f[i]$). Priors for the fecundity parameter $a_{d,Sb}$ were assigned a gamma distribution, fitted to the mean and standard deviation of estimates inferred from the abattoir data. For cattle, the prior for the density-dependent parameter (b_C) was given a normal

distribution, described by the mean and standard deviation of the estimates truncated at the upper limit of the 95% credible interval. Due to the low number of individuals in the abattoir dataset used to estimate the density-dependent parameter in small ruminants and the increased uncertainty around this parameter, a uniform distribution was applied to b_G and b_S, based on the 95% credible intervals estimated.

Bayesian approach to estimating mean worm burden and dispersion parameters by genotype for the human population

An additional Bayesian model was developed to estimate the worm burden of each genotype in each individual in the human study at baseline and thus the mean worm burden of each genotype ($m_{H,j}$) and dispersion parameter κ_H for the human population. A schematic of the Bayesian model is given in Figure S3.

Following the framework used for the dynamic model, the worm burden w_j of each genotype j in each individual i in the human dataset is assumed to be drawn from the population-level negative binomial (NB) distribution:

$$w_j\left[i\right] \sim NB\left(m_{H,j}, \kappa_H\right) \qquad [31]$$

The mean worm burden parameter was assigned a uniform prior distribution, $m_{H,j}$~Unif(0.001,100). This range covers and extends well beyond the worm burdens estimated in other modelling studies using high prevalence/pre-MDA datasets of urogenital schistosomiasis (*17*, *19*) so is weakly informative. The dispersion parameter was also assigned a weakly informative prior of κ_H~Unif(0.01,1), based on the observed overdispersion of the egg counts in the human dataset (Figure S4) and the dispersion parameters previously utilised when fitting schistosomiasis egg shedding data to a negative binomial distribution of worm burden (*16–18*, *20*).

The number of female worms of each genotype in each host individual, $w_{F,j}$, was then defined as a binomial sample from the worms of that genotype in that host with probability equal to the

average proportion of worms that are female (q_H), with remaining worms designated as male ($w_{M,j}$):

$$w_{F,j}\left[i\right] \sim Binom\left(q_H, w_j\left[i\right]\right) \tag{32}$$

The number of worm pairs of each combination was then determined by the number of available worms of each genotype and sex, with pairings assumed to take place following the same mating order as given above.

The observed (or where missing, imputed) set of typed miracidia of each category (μ1:6) in each positive individual ($om_{\mu 1:6}[i]$) were then described by a multinomial distribution:

$$om_{\mu 1:6}\left[i\right] \sim Multinomial\left(p_{\mu 1:6}\left[i\right], n\left[i\right]\right) \tag{33}$$

Where $n[i]$ is the number of typed miracidia in an individual host and $p_{\mu 1:6}[i]$ represents the multinomial probability of each miracidia in that host being of each of the six distinguished miracidia genotypes μ in the human population as described in Table S1 ($p_{\mu 1:6}=p(\mu 1), p(\mu 2)...p(\mu 6)$ where 1:6= *S. haematobium*, Hybrid1, Hybrid2 F1, Hybrid2 Backcross, Hybrid3 F1, Hybrid3 Backcross). The observed egg count per 10ml for each individual in the data ($oe[i]$) was then assumed to be a sample from a Poisson distribution with mean defined as the sum of the expected egg counts of all miracidia genotype in the individual:

$$oe\left[i\right] \sim Poisson\left(ee_{tot}\left[i\right]\right) \tag{34}$$

$$ee_{tot}\left[i\right] = \sum_{\mu=\mu 1,\mu 2...\mu 6} ee_{\mu}\left[i\right] \tag{35}$$

The expected egg count for each genotype is then a function of the number of couples of each pairing which produces miracidia of type μ in each individual ($c_{\mu}[i]$), the number of eggs per pair per 10ml given previously (a_H), and the per-pair density-dependent effect f[i], which is defined following the negative exponential:

$$ee_{\mu}\left[i\right] = c_{\mu}\left[i\right] * a_{H} * f\left[i\right] \qquad [36]$$

$$f\left[i\right] = e^{-b_{H}*(w_{tot}\left[i\right]-1)} \qquad [37]$$

Where w_{tot} represents the total worm burden of the individual. The probability $p_{\mu}[i]$ for each genotype is then determined by the relative proportion expected eggs of each genotype:

$$p_{\mu}\left[i\right] = ee_{\mu}\left[i\right] / ee_{tot}\left[i\right] \qquad [38]$$

Bayesian model implementation and comparison

All Bayesian analyses were carried out in R v3.5.1 (https://www.r-project.org). Bayesian simulations were run using JAGS version 4.3.0 using Markov Chain Monte Carlo (MCMC) simulations (two chains, 200,000 iterations, burn-in of 5000, thinning interval of 40) implemented using the 'rjags' and 'coda' packages.

Model convergence was assessed visually and using the "gelman.diag" function in the coda package (*21*, *22*). The upper confidence interval for the potential scale reduction factor was less than 1.1 for all parameters estimated and the multivariate potential scale reduction factor was less than 1.1 for all Bayesian frameworks implemented here, indicating no concerns regarding convergence (*23*).

The four models for alternative formulations of the density-dependent function in livestock (equations 16-20) which were applied to the abattoir data were compared using Deviance Information Criterion (DIC). This was calculated using the dic.samples function in the package 'rjags' (*24*).

Estimating dynamic model transmission parameters: livestock

S. bovis transmission dynamics within and between the livestock populations were assumed to be at equilibrium at the time of data collection. This assumption is supported by the fact that *S. bovis* has been reported in the Senegal River Basin since the 1960s (*25*, *26*), with no interventions systematically targeting the animal population nor any systematic mollusciciding in this area.

Following this assumption the larval stage to worm transmission rate ($A_{d,Sb}$) in each species can be estimated by setting the left hand side of equation 1 to zero and using the estimated values for the worm death rate (σ_d) and the equilibrium values of mean worm burden ($\hat{m}_{d,Sb}$) and larval *S. bovis* pool ($\hat{L}_{Sb}$):

$$A_{d,Sb} = \frac{\hat{m}_{d,Sb}\left(\sigma_d\right)}{\hat{L}_{Sb}} \qquad [39]$$

For consistency and to enable broad comparison with the estimated transmission rates from the infectious pool to human hosts, L_{Sb} at stability was assigned a value of 0.16, representing the proportion of the cercarial pool shed by *Bulinus* spp. which was estimated to comprise of *S. bovis,* based on genotyped cercariae data from the malacology surveys described in (*1*).

Schistosomiasis transmission models generally assume that the force of infection on the snail population is a linear function of egg excretion by the definitive host population (*5*, *16*). This assumption is supported by early experimental work (*27*) and has been utilised in multi-host *S. japonicum* systems (*28–30*) to estimate the relative contribution of each host species to transmission.

By extending this to assume that the contribution of each species to the environmental reservoir of infectious material is proportional to the egg shedding by that population, equation 2 can be rearranged to give a general transmission rate $\beta_{All,Sb}$, constant for all species which shed *S. bovis*:

$$\frac{dL_{Sb}}{dt} = \left[\beta_{All,Sb} \sum_{d=C,\,S,\,G} \psi_{d,\,Sb} \lambda_{d,Sb} \bar{f}_d N_d\right] - \gamma\, L_{Sb} \qquad [40]$$

Following this assumption, and with the assumption of dynamic equilibrium at the time of sampling, the transmission parameter $\beta_{All,Sb}$ can be estimated by setting the left hand side of the equation to zero:

$$\beta_{All,Sb} = \frac{\gamma \hat{L}_{Sb}}{\sum_d \hat{\psi}_{d,\,Sb} \lambda_{d,Sb} \hat{f}_d N_d} \qquad [41]$$

The transmission parameter $\beta_{All,Sb}$ can then be estimated using equilibrium values of the *S. bovis* larval pool ($\hat{L}_{Sb}$ as given previously) and the mean number of *S. bovis* pairs for each species ($\hat{\psi}_{d,\,Sb}$), together with estimated values of the decay rate of infectious material (γ), daily egg shedding by a mated pair ($\lambda_{d,Sb}$), population size (N_d), and the mean per female density-dependent reduction in fecundity at equilibrium ($\hat{f}_d$), which is estimated by applying the equilibrium values of mean worm burden $\hat{m}_{d,Sb}$, the dispersion parameter $\hat{\kappa}_d$ and the estimated sex ratio q_d in each species to equation 5.

Estimating dynamic model transmission parameters: human population

The component of the total larval pool (L_{tot} in the dynamic model) that encompasses schistosome genotypes which are shed by the human population is given as L_{H}, and the proportion of this infectious pool that is comprised of each genotype (μ1:6 in Table S1) is assumed then to be proportional to the corresponding miracidia shedding by the human population. This means that the transmission parameter β_H is constant for all genotypes. This assumption was validated against the observed distribution of cercariae genotypes identified from the snail survey described in (*1*) where no significant difference was found between the proportions of total analysed miracidia of each genotype shed by the human population and corresponding proportions of analysed cercariae (see also Table S4).

It is also assumed that the transmission dynamics were at equilibrium at the time of the data collection. This assumption is supported by the fact the current control interventions targeting the human population, comprising mass drug administration (MDA) of praziquantel to school-aged children, have been in place since 2006/7, and the fact that no significant difference was observed in the schistosomiasis prevalence nor proportion of children shedding different schistosome genotypes between the 2016 and 2017-18 surveys described in (*1*).

Following these assumptions and rearranging equation 2, the transmission parameter β_H can be estimated in the same way as for livestock:

$$\beta_H = \frac{\gamma \hat{L}_H}{\sum_j \hat{\psi}_{H,j} \lambda_{H,j} \hat{f}_H N_H} \quad [42]$$

Here the density of infectious material at stability (denoted $\hat{L}_H$) is given as the observed proportion of the cercarial pool comprising genotypes of human origin (0.84 in *1*), enabling transmission rates from this pool to human hosts and from the equivalent pool of animal origin (L_{Sb}) to human hosts, to be compared.

Similarly, by setting the left hand side of equation 1 to zero, the larval stage to worm transmission rate can be estimated for each worm genotype in the human population using the estimated equilibrium values of mean worm burden and density of infectious material ($\hat{m}_{H,j}$ and $\hat{L}_j$) for each genotype *j,* and using the estimated equilibrium worm death rate ($\hat{\sigma}_H$) based on assumed treatment coverage levels:

$$A_{H,j} = \frac{\hat{m}_{H,j}\left(\hat{\sigma}_H\right)}{\hat{L}_j} \quad [43]$$

Estimation of *R₀*

For macroparasites, R_0 is defined in Anderson & May (*16*) as the average number of mated female offspring produced during the lifespan of one mated adult female in the absence of constraints on population growth. If the value of R_0 is less than 1, i.e. the average female worm does not replace herself in the next generation, the parasite cannot be maintained in the population. The formulation of R_0 for schistosomes is given as:

$$R_0 = qT_1T_2\phi \quad [44]$$

With T_1 representing the transmission from snail to mammalian host, T_2 transmission from mammalian host to snail, *q* the probability of worm being female, and ∅ the probability a given female worm is mated. Adapting this definition to the transmission framework described

above, B_d can be used to denote the per pair daily transmission rate from a mated female in host species *d,*

$$B_d = \beta_d \lambda_d \qquad [45]$$

The life expectancy of the average female worm in days is given by $\sigma_d{}^{-1}$, therefore the transmission rate T_2 for each species would be given as:

$$T_2 = \frac{B_d\ e^{-b_d}}{\sigma_d} \qquad [46]$$

The value e^{-b_d} is included as the per female density dependent reduction in a host with *w* worms being given by $e^{-b_d(w-1)}$, and in the case of single mated female the value for *w* would be two (one male one female).

The transmission rate T_1, which can be defined here as the rate of transmission from the pool of infectious material to the definitive host population *d* can similarly be given as:

$$T_1 = \frac{A_d N_d}{\gamma} \qquad [47]$$

Where γ^{-1} is the average survival time of infectious material in the environment.
Due to the complexity of the mating function (equations 9-13) a mating probability cannot be given for the general case. Furthermore, in most endemic settings where worm burdens are overdispersed in the host populations, and have an unequal sex ratio in favour of males (as has been observed in this system) most hosts harbouring a female worm will also harbour at least one male worm. Therefore, in calculating the R_0 for schistosomes within a mean worm burden framework, it is often assumed that all female worms are mated ($\emptyset = 1$) (*4*, *17*). The R_0 for a transmission system involving a single host species *d* would then be given by:

$$R_0{}^d = \frac{A_d N_d B_d\ e^{-b_d}}{\sigma_d \gamma} q_d \qquad [48]$$

Estimation of the R_0 for the Hyb genotype in the human population was used to evaluate the capacity of *S. haematobium x S. bovis* hybrids to be maintained in the human population in the absence of zoonotic spillover. If the average "*Hyb*" female does not replace herself in the next generation (R_0<1) this genotype category could not be maintained without the creation of novel hybrids via zoonotic spillover. Therefore $R_0^{H,Hyb}$ was estimated:

$$R_0^{H,Hyb} = \frac{A_{H,Hyb} N_H B_H \; e^{-b_H}}{\sigma_H \gamma} q_H \qquad [49]$$

And for comparison, $R_0^{H,Sh}$ was also calculated in the same way (utilising the transmission rate $\boldsymbol{A_{H,Hyb}}$).

Partitioning of R_0 for the multi-host *S. bovis* system

The formulation for a single host species R_0^d given in equation 48 was extended to the multi-host *S. bovis* system to calculate the overall R_0 across the entire host species community, denoted $R_0^{tot,Sb}$. The next generation matrix approach described by Roberts and Heesterbeek (*31*) was applied, where the "next generation" here refers to the transmission of infection from a definitive host to the environmental pool of infectious material and subsequent successful infection of another definitive host and generation of a new female worm. In a system with *n* definitive host species, the next generation matrix (*K*) would be constructed as an *n x n* matrix, with each element $k_{d,g}$ representing the expected number of female worms in definitive host species *d* that would be generated during the lifetime of a mated female worm in host population *g*. The diagonal elements of the matrix (where *d*=*g*) are therefore the within-species $R_0^{d,Sb}$.

The next generation matrix for the three host species involved in *S. bovis* transmission in the study region can then be given by:

$$K = \begin{matrix} k_{CC} & k_{CG} & k_{CS} \\ k_{GC} & k_{GG} & k_{GS} \\ k_{SC} & k_{SG} & k_{SS} \end{matrix} = \begin{bmatrix} \frac{A_C N_C \mathrm{B}_C e^{-b_C}}{\sigma_C \gamma} q_d & \frac{A_C N_C \mathrm{B}_G e^{-b_G}}{\sigma_G \gamma} q_d & \frac{A_C N_C \mathrm{B}_S e^{-b_S}}{\sigma_S \gamma} q_d \\ \frac{A_G N_G \mathrm{B}_C e^{-b_C}}{\sigma_C \gamma} q_d & \frac{A_G N_G \mathrm{B}_G e^{-b_G}}{\sigma_G \gamma} q_d & \frac{A_G N_G \mathrm{B}_S e^{-b_S}}{\sigma_S \gamma} q_d \\ \frac{A_S N_S \mathrm{B}_C e^{-b_C}}{\sigma_C \gamma} q_d & \frac{A_S N_S \mathrm{B}_G e^{-b_G}}{\sigma_G \gamma} q_d & \frac{A_S N_S \mathrm{B}_S e^{-b_S}}{\sigma_S \gamma} q_d \end{bmatrix} \qquad [50]$$

The formulation of the next generation matrix given here assumes no spatial structuring of transmission. This assumption is justified by the observation that households keeping cattle,

sheep and goats were found in all of the study villages and that these species generally share common water access points. The $R_0^{tot,Sb}$ was then calculated as the dominant eigenvalue of the transmission matrix K using the 'eigen' function in R v3.5.1 (*29*, *32*).

Longitudinal simulations: Predicting the impact of eliminating zoonotic transmission under current levels of MDA and under scenarios of enhanced MDA coverage

Longitudinal simulations of the dynamic model were implemented to examine the potential impact of removing zoonotic transmission under current levels of treatment coverage in the human population, and also under enhanced treatment coverage levels. Annual mass drug administration (MDA) treatment coverage of school-aged children in the study area was assumed to be 50% at baseline, based on the data provided by the preventative chemotherapy databank of the World Health Organisation (WHO) for endemic regions of Senegal (*7*).

The impact on the burden of each worm genotype (and total worm burden) was simulated under treatment coverage levels of 50% (assumed baseline) and increased coverage levels of 75% and 90%, both with current assumed levels of zoonotic transmission ($A_{H,Sb}$ estimated from Bayesian output) and in scenarios where there is no zoonotic transmission (simulated by setting $A_{H,Sb}$ to zero). The proportion of the total mean worm burden that is comprised of hybrids was calculated within each simulation. For all scenarios simulated here, efficacy of praziquantel is assumed to be 95% (*58*) and it is assumed that *S. bovis* continues to circulate within the animal population at equilibrium, with L_{Sb} therefore assumed to be stable.

Longitudinal simulations: Predicting the impact on transmission dynamics of interventions targeting the animal population

Cattle were identified as both essential and maintenance hosts for *S. bovis* transmission (see results main text), and therefore the impact on $R^{tot,Sb}$ by annually treating cattle with praziquantel was explored, simulating coverage rates of 0-100%. Given concerns regarding the practicalities and potential negative consequences of treating livestock with praziquantel (*33*) the impact on $R_0^{tot,Sb}$ of removing 0-100% of the cattle population from schistosomiasis transmission was also evaluated. An alternative intervention that could theoretically achieve this could be provision of water troughs which are used as the sole source of water for a subset of the cattle population.

Longitudinal dynamic model simulations were also carried out to assess the impact of annually treating the cattle population on the mean worm burden of each livestock population, and on

the density of infectious *S. bovis* material in the environment, which has been identified as the source of zoonotically-acquired *S. bovis* infections in humans. Treatment efficacy for all simulations was assumed to be 95% (*34*), and treatment coverage rates of 25%, 50% and 75% were applied to the longitudinal simulations.

Treatment coverage was simulated by adding a worm death rate due to treatment parameter to the worm death rate parameter σ_C. Removal of cattle from the population that can effectively contribute to transmission was simulated by reducing N_C by a factor of 0-100%.

Model implementation and uncertainty analysis.

In order to account for the uncertainty in the Bayesian estimation for key parameters in the human population (the dispersion parameter, κ_H, and mean worm burdens for each genotype in the human population $m_{H,j}$), one posterior MCMC chain was randomly selected (5,000 iterations after discarding of burn-in period and thinning), and the transmission parameters $A_{H,j}$ and B_H calculated for each iteration. These 5,000 estimations of each transmission parameter were then used to generate 5,000 estimations of $R_0{}^{H,Hyb}$. Similarly, for each iteration the estimated worm burdens were used to estimate expected mean couples of each pairing combination, and the proportion of total couples that would be comprised of each pair combination following the assumptions regarding mating described earlier.

Due to the uncertainty around many of the parameter estimates for the livestock component of the model, which were derived from the data, literature and from government resources, these were assigned a distribution of plausible values for each host species for the estimation of transmission rates and R_0. A random set of values for the population estimates (N_d), death rate of worms (σ_d), decay rate of infectious material in the environment (γ) and daily mass of faecal production for each species (g_d) were generated using the Latin Hypercube sampling method (LHS) to efficiently sample the parameter space (*80,81*), drawing from distributions given in Table S2 to generate 1000 sets of parameters.

The LHS method was not suitable for exploring the parameter space for the values for mean worm burden, worm dispersion, fecundity and density-dependence ($m_{d,Sb}$, κ_d, $a_{d,Sb}$ and b_d) as these were jointly estimated within the Bayesian framework described (the LHS method, by selecting independent values for each variable, would not account for the covariance between

parameter estimates). Therefore, to account for the uncertainty in these parameter estimates, 1000 iterations containing estimates for these four parameters were randomly selected from one posterior MCMC chain (following discarding of burn-in period) for each of the livestock host species.

Thus 1000 sets of all parameters were created and used to generate 1000 values of $\beta_{All,Sb}$, $\lambda_{d,Sb}$, $A_{d,Sb}$, $R_0^{d,Sb}$ and $R_0^{tot,Sb}$, and to calculate the median and 95% confidence intervals for these estimates (where 95% confidence intervals here refer to the 95-centile range).

For all longitudinal simulations 1000 sets of parameter values were applied to create 1000 simulations. These were generated as described above for parameters relating to the livestock population. For parameters relating to the human population, 1000 iterations were randomly chosen from one posterior MCMC chain from the Bayesian analysis output, with transmission parameters estimated for each. Median values are presented and 95 centiles given as 95% confidence intervals.

The dynamic models were implemented in R v3.5.1 (https://www.r-project.org) using the "desolve" package v1.21 (*82*), with the function "lsoda" implemented within this package to solve the ordinary differential equations. The "lhs" package was used for creating the latin hypercube in the uncertainty analysis (*83*).

Supplementary Text: Additional results.

Bayesian estimation of worm burden parameters in human population

The median posterior estimate for the overall aggregation parameter for worms in the human population (κ_H) was 0.33 (95% BCI 0.29-0.39; mean 0.34 S.D 0.03). This low value is as expected due to the observed overdispersion of observed egg count data (Figure S4).

In order to validate the worm burden estimates from the Bayesian analysis and assumptions regarding mating order, the expected percentages of total couples in the human population that would be comprised of each pair combination (these proportions can be considered as the expected proportion of miracidia output of each category from humans) were compared to the observed proportions of miracidia and cercariae of each genotype (Table S4 and Figure S5). The observed miracidia genotype percentages fall within the 95% confidence intervals for the corresponding worm-pair estimates generated by the Bayesian output.

Evidence for density-dependent reduction in schistosome fecundity

Worm count data from post-mortem of livestock abattoir specimens is plotted in Figure S6, showing skewed worm count distributions within worm-positive animals (small ruminant data were pooled due to the low number of worm positive individuals). The worm count data also revealed a biased sex ratio in favour of male worms, with 61% of all worms collected identified as male.

Evidence for density-dependent reduction in schistosome fecundity in cattle, sheep and goats infected with *S. bovis,* based on abattoir worm counts and with MHT results as a proxy for egg counts, is summarised in the main text. Worm count data is plotted against per-pair per-gram estimated egg count data in Figures S7 (cattle) and S8 (small ruminants).

Bayesian estimation of model parameter values for livestock

The values of $a_{d,Sb}$ and b_d for livestock estimated from the abattoir data are summarised in Table S5, including the Deviance Information Criteria (DIC) used for model comparison. The model with the lowest DIC for the cattle data was the exponential model with density-dependence proportional to total worm burden (equation 18). For small ruminants, there was very little variation in the penalized deviance, although estimates were based on a lower number of data points. The exponential model with density-dependence as a function of total worm burden was therefore applied to the live animal data using priors generated from the abattoir data.

The median estimates for the dispersion parameter in all species were consistent with an overdispersed distribution of worm burden (κ_d<1 for all) and the estimated daily egg shedding by a *S. bovis* worm pair in the absence of density-dependent effects ($\lambda_{d,Sb}$) was very similar in all species (see Table S2) with overlapping confidence intervals. However, the median estimated value for mean worm burden ($m_{d,Sb}$) in cattle was much higher (46.41, 95% BCI 31.83-72.03) than for goats (1.11, 95% BCI 1-1.68) or sheep (1.18, 95% BCI 1.01-5.34).

Impact of simulations targeting the cattle population

The predicted impact on $R_0^{tot,Sb}$ of treating the cattle population annually or reducing the size of the cattle population involved in transmission is described in the main text. The impact of annual treatment of cattle is further explored in the longitudinal simulations shown in Figure S9. These simulations predict that even if only 25% of cattle were treated annually, over 10 years this could reduce the mean worm burden in cattle to less than a quarter of the current estimate (median estimated mean worm burden in cattle at 10 years, $m_{C,Sb}$, 11.11; 95% CI 5.77-27.74). If the zoonotic risk to the human population is considered the main outcome of interest (as opposed to disease burden in the livestock population), the estimated impact of interventions on reducing this zoonotic source of infection can be inferred from the relative reduction in the environmental source of *S. bovis* infection, defined in the model as L_{Sb}. Here again, the simulations predict that even treating a relatively low proportion (25%) of the cattle annually would reduce this environmental source of infection by nearly 50% within 5 years (median estimate: 54%; 95% CI 47-66%, 5 years of treating 25% of cattle annually).

For higher treatment coverage levels of 50%, median estimates from simulations predict a more dramatic decrease in livestock worm burden and environmental *S. bovis* reservoir within 10 years, although considerable uncertainty remains (median $m_{C,Sb}$: 1.5, 95% CI 0.5-7.1; median L_{Sb} relative to baseline: 3.5%, 95% CI 1.1-15.5%). At 75% treatment coverage levels, simulations predict a near-elimination scenario such that the zoonotic risk to humans would be expected to be minimal (median $m_{C,Sb}$: 0.1, 95% CI 0.02-1; median L_{Sb} relative to baseline: 0.13%, 95% CI 0.01-1.9%).

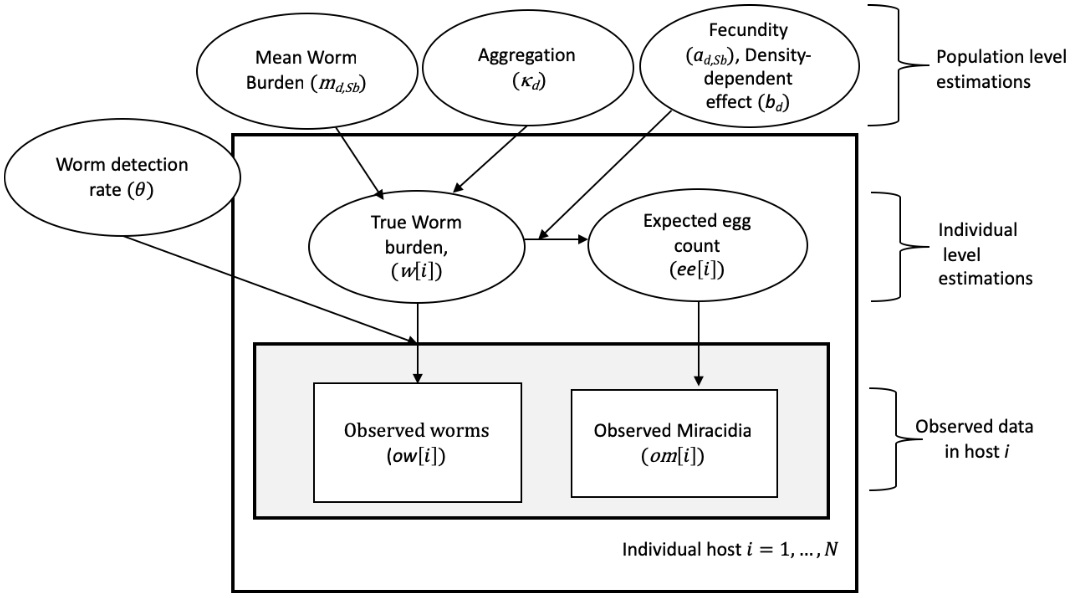

Figure S1. Schematic representing the Bayesian model for estimating fecundity and density-dependent parameters for *S. bovis* is livestock from abattoir data.

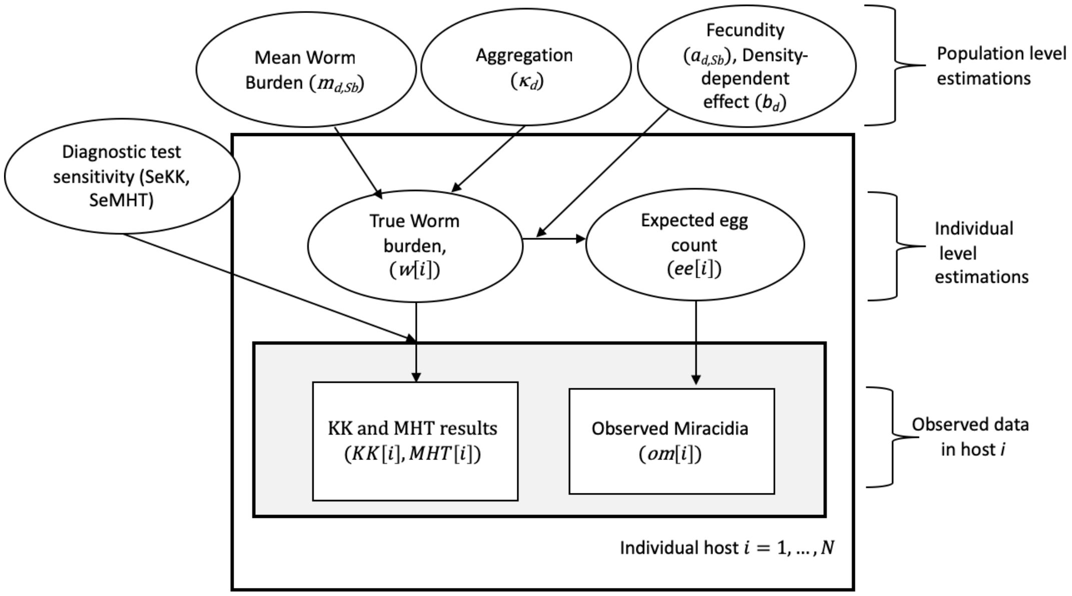

Figure S2. Schematic representing the Bayesian model for estimating mean worm burden and dispersion parameters for *S. bovis* in livestock, using live animal data and applying priors from abattoir data.

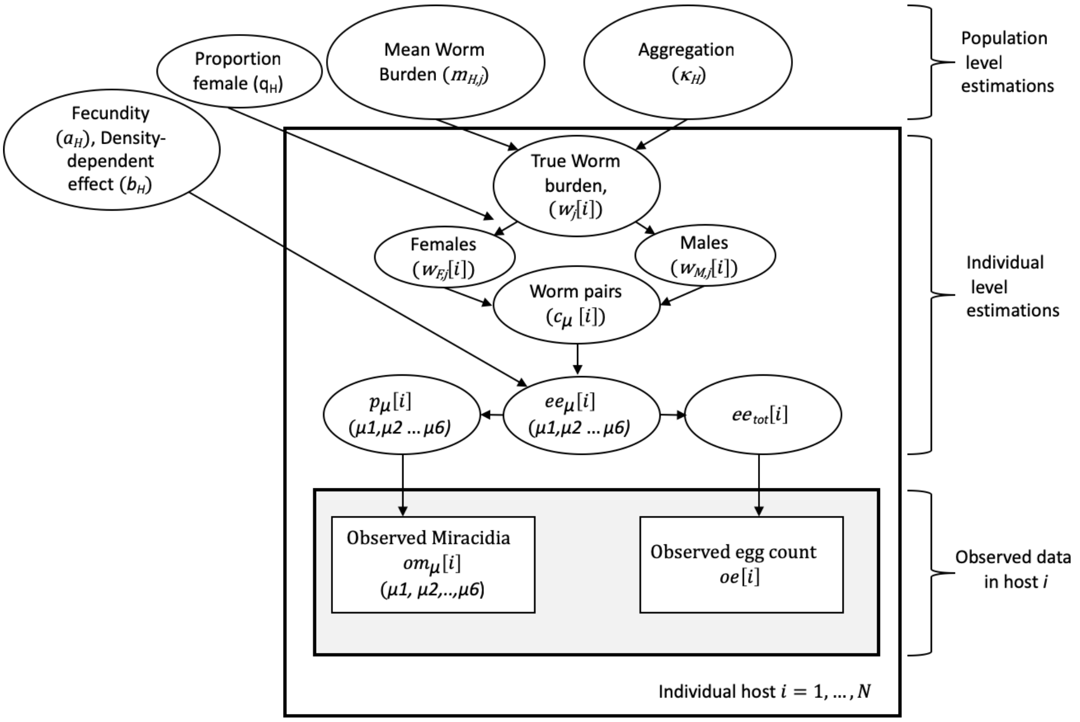

Figure S3. Schematic representing the Bayesian model for estimating mean worm burden ($m_{H,j}$) and dispersion (κ_H) of haematobium group schistosome genotypes in the human population using observed subset of genotyped miracidia and observed mean egg count per 10ml urine.

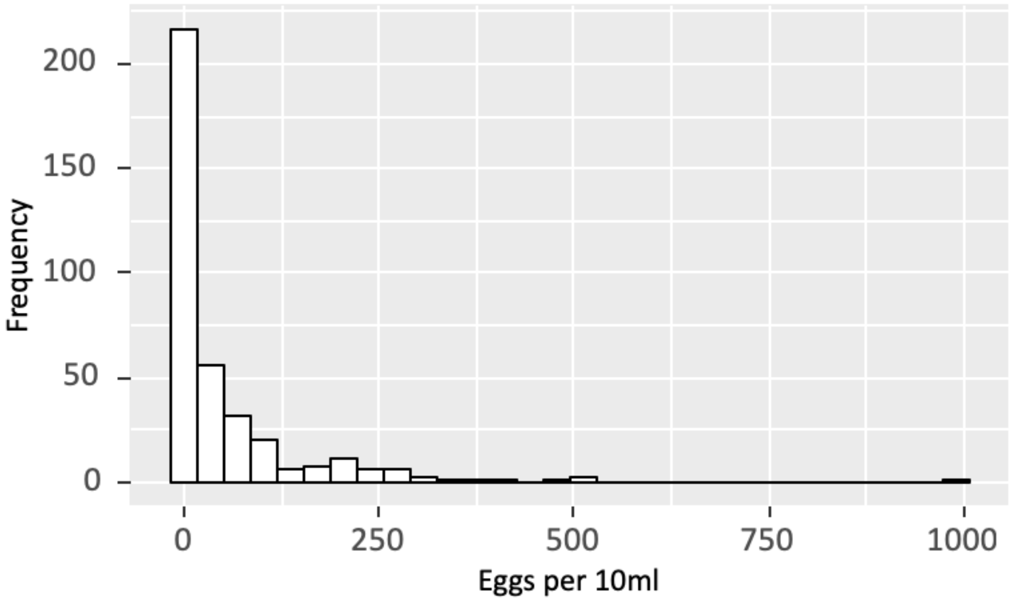

Figure S4. Frequency histograms showing mean egg count per 10ml (urine), Richard Toll/Lac de Guiers human survey (children aged 5-17), 2016. n=375.

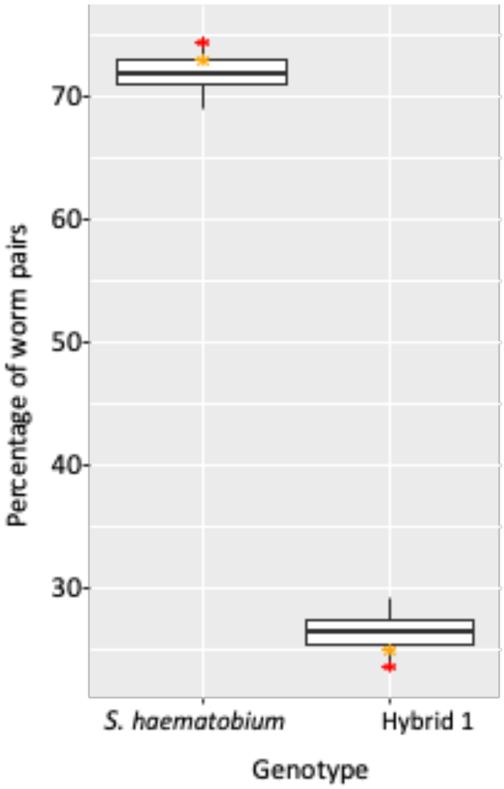

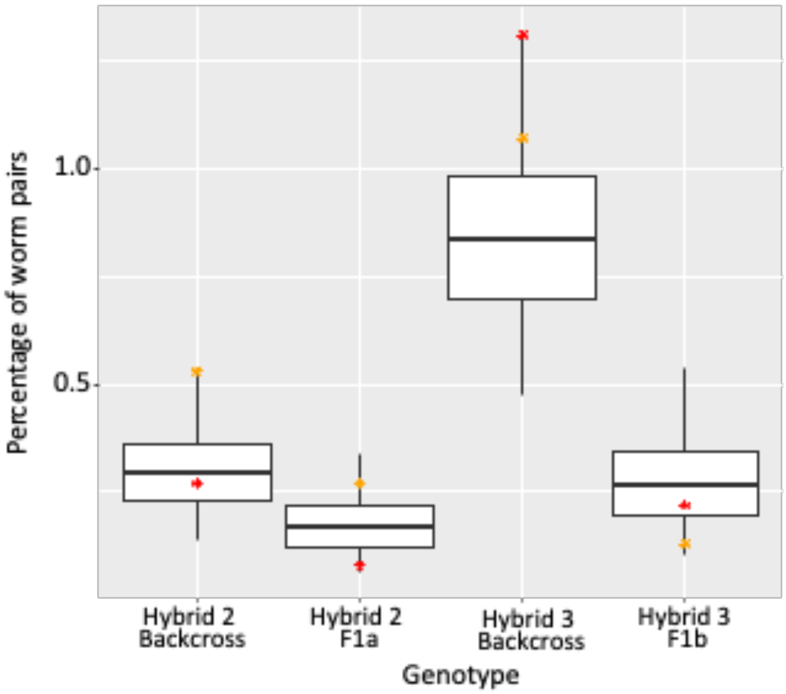

Figure S5. Estimated percentage of all worm pairs in the human population that will produce miracidia of each genotype based on Bayesian estimation of mean worm burden. Orange star indicates observed percentage of typed miracidia of each genotype, and red star indicates estimated percentage of typed miracidia, weighted by egg shedding intensity of individual host. (95% confidence intervals derived from uncertainty analysis).

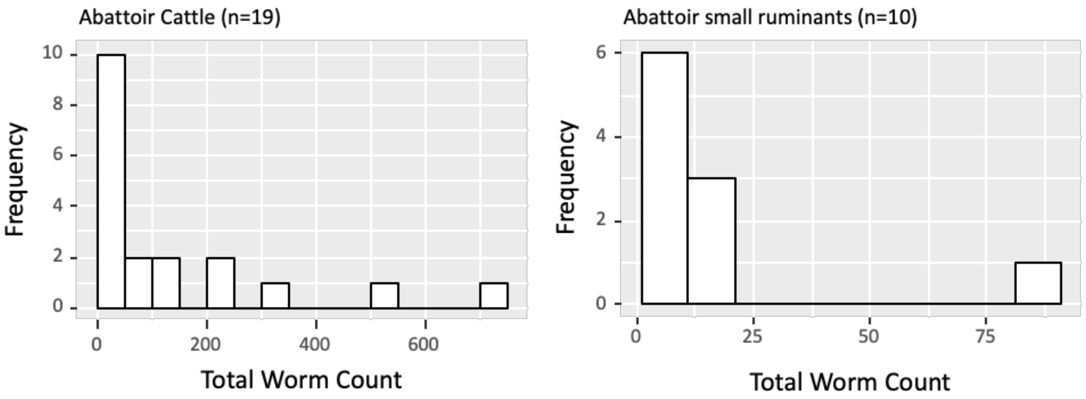

Fig S6. Frequency histograms showing worm count in livestock abattoir specimens infected with *Schistosoma bovis*.

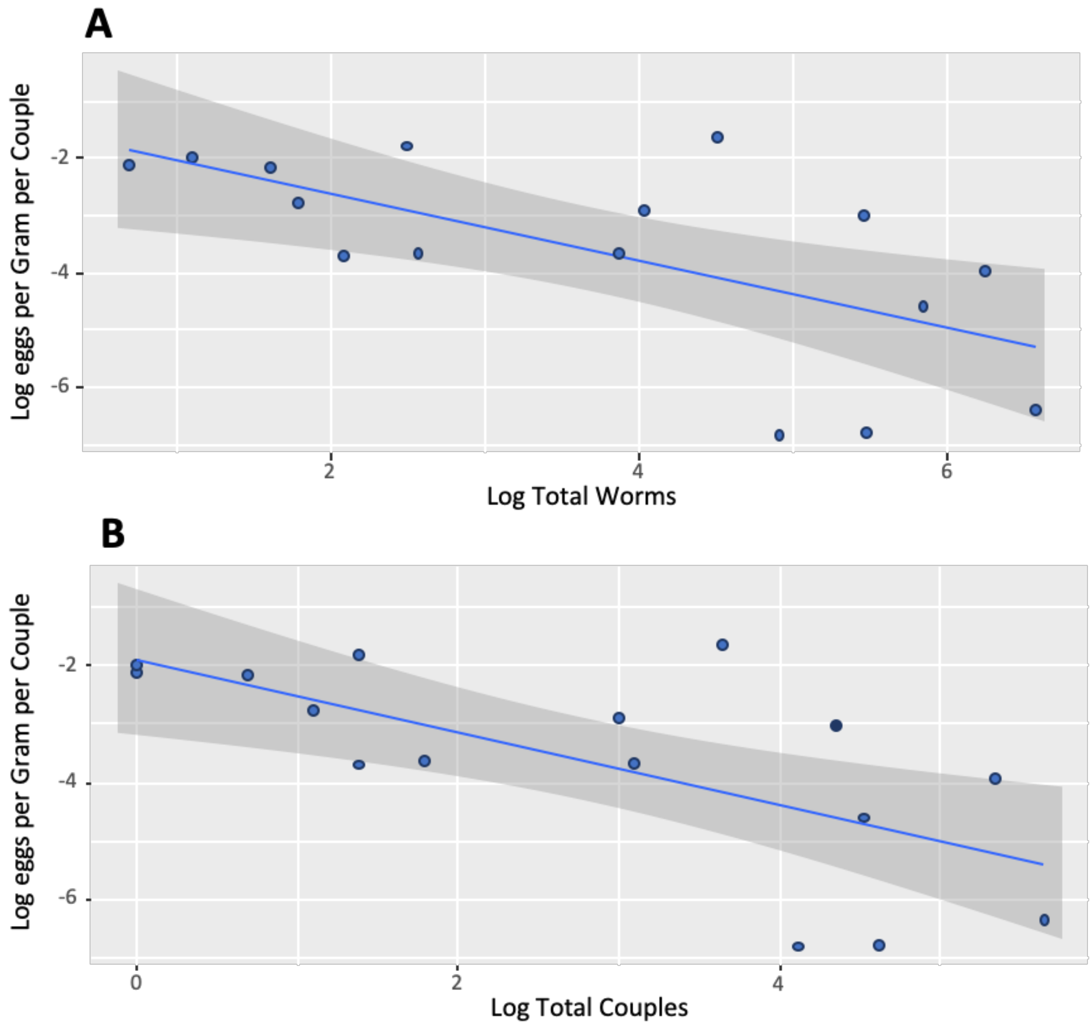

Figure S7. Estimated egg count per gram of faeces per *Schistosoma bovis* worm pair in post-mortem cattle specimens compared to observed total worm count (A) and total worm pair count (B).

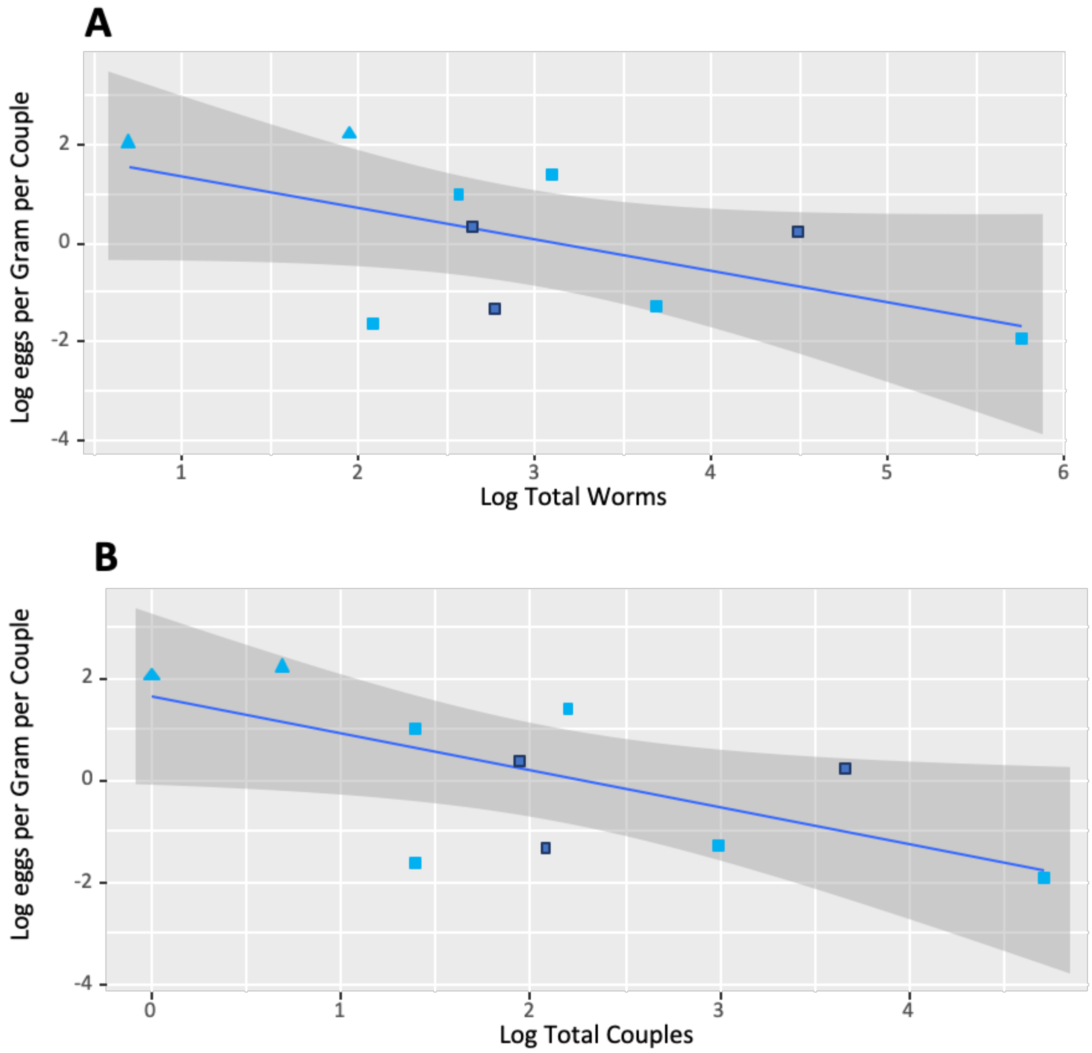

Figure S8. Estimated egg count per gram of faeces per worm pair in post-mortem sheep (triangles) and goat (squares) specimens compared to observed total worm count (A) and total worm pair count (B). Animals infected with *Schistosoma bovis* only (dark blue) are distinguished from those infected with other schistosome genotypes (*S. curassoni, S. curassoni* hybrids and co-infected individuals: light blue).

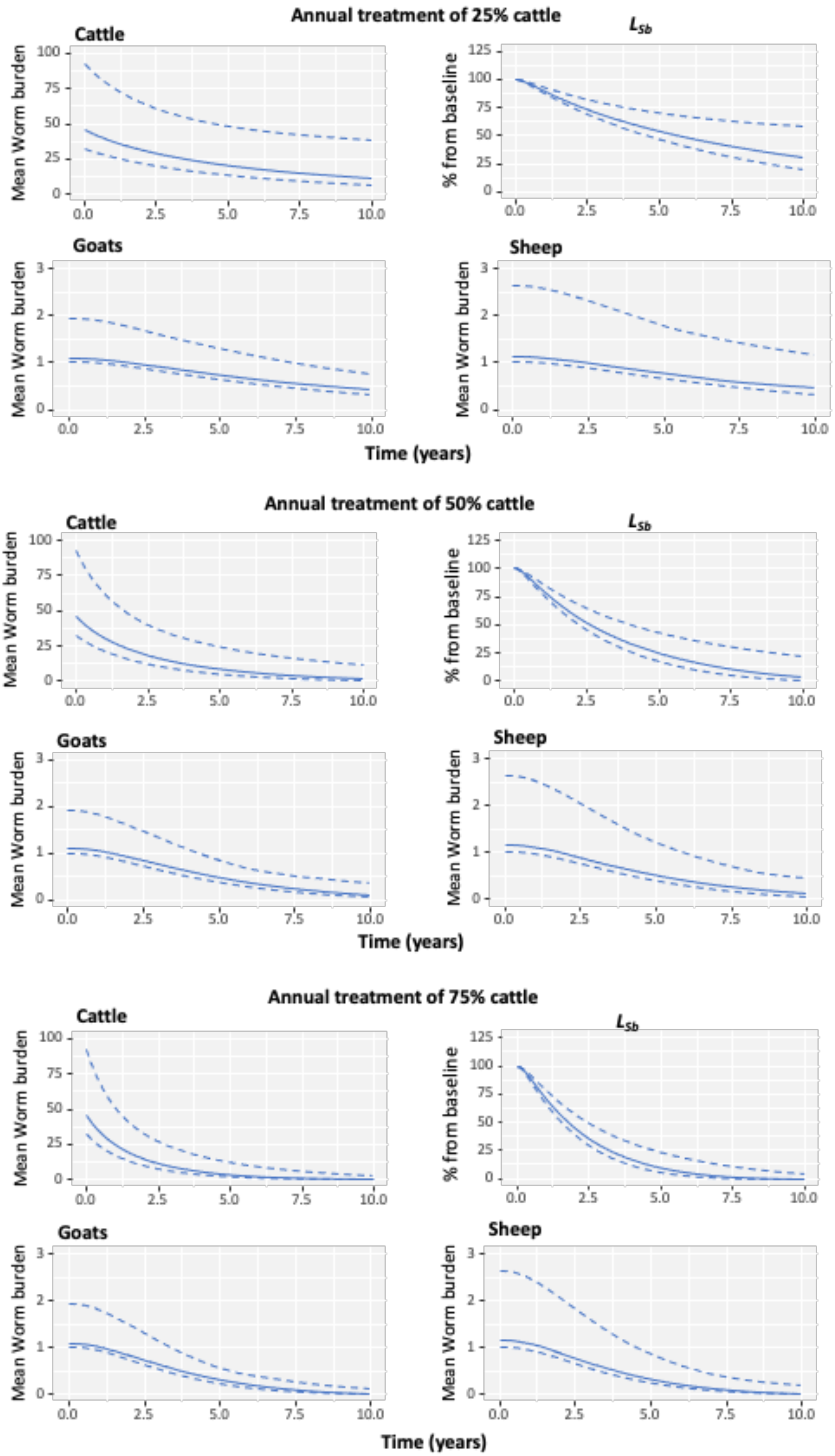
Annual treatment of 25% cattle
Cattle
L_{Sb}
Goats
Sheep
Mean Worm burden
% from baseline
Time (years)
Annual treatment of 50% cattle
Cattle
L_{Sb}
Goats
Sheep
Mean Worm burden
% from baseline
Time (years)
Annual treatment of 75% cattle
Cattle
L_{Sb}
Goats
Sheep
Mean Worm burden
% from baseline
Time (years)

Figure S9. The predicted impact of annually treating 25%, 50% or 75% of the cattle population in the Lac de Guiers region of Senegal, on the mean worm burden of *Schistosoma bovis* in livestock species and density of *S. bovis* environmental reservoir (L_{Sb}, % change from baseline). Treatment assumed to be 95% efficacious.

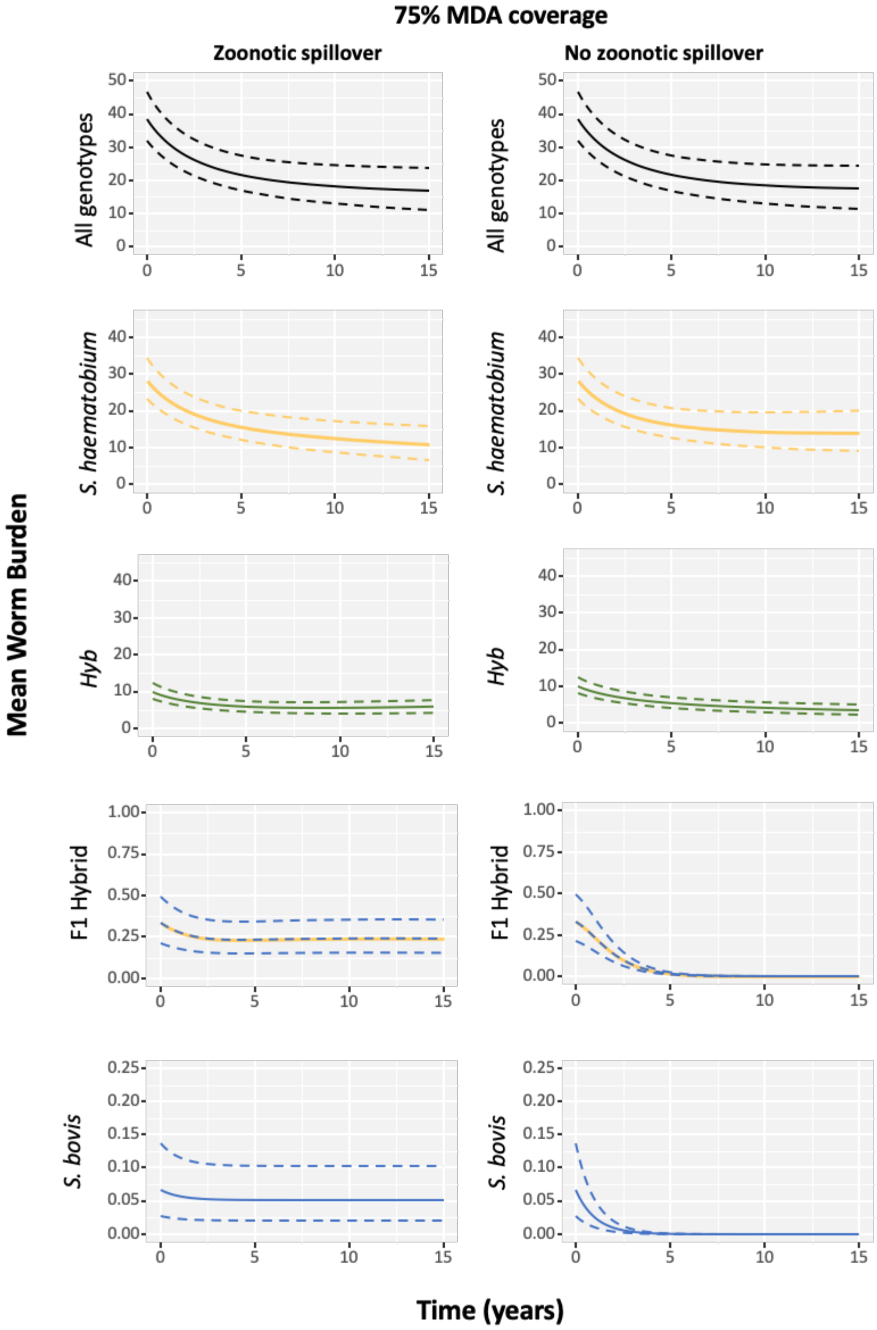

Figure S10. Longitudinal simulation under enhanced mass drug administration (MDA) coverage with praziquantel (75% coverage school-aged children): Mean worm burden in human population of *S. haematobium*, *Hyb*, F1 Hybrids and *S. bovis*; under current level of zoonotic transmission and with no zoonotic transmission; Median shown as solid lines, dashed lines show 95% confidence intervals.

**Definitive host**	**Miracidia/ cercariae profile (μ1:7)**	***cox1***	**ITS**	**Assumed parentage**	**Parental Worm pair**	**Component of larval pool (*Lj*)**
Human	1.*S. haematobium*	*Sh*	*Sh*	Female *Sh* & Male *Sh*	*i)* ψ_{H,Sh_Sh}	*Sh*
	2.Hybrid1	*Sb*	*Sh*	Female *Hyb* & Male *Sh*, or Female *Hyb* & Male *Hyb*	*ii)* ψ_{H,Hyb_Sh} *iii)* ψ_{H,Hyb_Hyb}	*Hyb*
	3. Hybrid2 Backcross[a]	*Sb*	*Mix ShxSb*	Female *F1a* & Male *Sh*	*iv)* $\psi_{H,F1a\_Sh}$	
	4. Hybrid3 Backcross[b]	*Sh*	*Mix ShxSb*	Female *Sh* & Male *F1b*	*v)* ψ_{H,Sh_F1b}	
	5.Hybrid2 F1[a]	*Sb*	*Mix ShxSb*	Female *Sb* & Male *Sh*	*vi)* ψ_{H,Sb_Sh}	*F1a*
	6.Hybrid3 F1[b]	*Sh*	*Mix ShxSb*	Female *Sh* & Male *Sb*	*vii)* ψ_{H,Sh_Sb}	*F1b*
Cattle Sheep Goats	7. *S. bovis*	*Sb*	*Sb*	Female *Sb* & Male *Sb*	*viii)* ψ_{C,Sb_Sb} *ix)* ψ_{S,Sb_Sb} *x)* ψ_{G,Sb_Sb}	*Sb*

[a] Hybrid2 Backcross and Hybrid2 F1 distinguished based on chromatogram peak height.
[b] Hybrid3 Backcross and Hybrid3 F1 distinguished based on chromatogram peak height

Table S1. Classification of observed miracidia and cercariae based on molecular profile and definitive host species observed to be shedding each genotype. Assumed worm parentage (ψ_{d,jF_jM}) based on *cox1* and ITS and corresponding component of larval pool (L_j) in dynamic model which each worm pair contributes to, where $j \in \{Sh, Sb, F1a, F1b, Hyb\}$.

Parameter Definitions, Estimation and Source			
**Notation**	**Description (units)**	**Estimate/distribution**	**Source**
N_d	Population estimates species *d;* humans=H, cattle=C, goats=G, sheep=S	N_H=15,000<br>N_C=Unif(38700,47300)<br>N_G=Unif(34200,41800)<br>N_S=Unif(38250, 46750)	(*35*)<br>(N_H refers to estimated population aged 5-18).
σ_d	Death rate of worms in host *d* (years)	σ_H=1.38^{-1}<br>σ_C= Unif(2.67^{-1},2^{-1})<br>σ_G= Unif(2.22^{-1},1.33^{-1}<br>σ_S= Unif(2.22^{-1},1.33^{-1})	(*7–9*)<br>(Varied in longitudinal simulations).
γ	Loss rate, infectious material (days)	γ= Unif(120^{-1}, 60^{-1})	(*36*)
$q_{d,}$	Proportion of worms of that are female in host *d.*	0.4	Estimated from data
g_d	Daily faecal output for livestock host species *d* (grams)	g_C= Unif(2300,3000)<br>g_G = Unif(200, 330)<br>g_S= Unif(200, 330)	(*37–40*)
$a_{d,Sb}$	*S. bovis* fecundity parameter host species *d* (Eggs/worm pair/gram faeces)	See Table S6	Estimated from data
a_H	Eggs/worm pair/10ml urine in human host	5.2	(*17*)
b_d	Density-dependent effect on per pair fecundity	b_H = 0.05<br>Livestock see Table S6	Humans:(*17*)<br>Livestock: Estimated from data
κ_d	Aggregation parameter in definitive host *d*	κ_H= 0.33<br>(BCI 0.29, 0.39)<br>Livestock See Table S6	Estimated from data
β_H	Transmission rate from eggs/miracidia in the human population to the pool of infection material L_H (days)	$2.19\text{x}10^{-10}$<br>(BCI: 1.97×10^{-10}, 2.47×10^{-10})	Estimated from data
$\beta_{All,Sb}$	Transmission rate from eggs/miracidia in a definitive host to the pool of infection material L_{Sb} (days)	1.61×10^{-11}<br>(BCI: 9.86×10^{-12}, 2.57×10^{-11})	Estimated from data.
$A_{H,j}$	Transmission rate of larval pool L_j to human population (days)	$A_{H,Sh}$ = 0.09<br>(BCI: 0.08, 0.11)<br>$A_{H,Hyb}$ = 0.09<br>(BCI: 0.07, 0.10)<br>$A_{H,F1a}$ = 0.17<br>(BCI: 0.06,0.53)<br>$A_{H,F1b}$ = 0.19<br>(BCI: 0.07,0.55)<br>$A_{H,Sb}$ = $8.3\text{x } 10^{-4}$<br>(BCI: 3.2×10^{-4}, $1.8\text{x}10^{-3}$)	Estimated from data
$A_{d,Sb}$	Transmission rate from larval pool L_{Sb} to definitive host population *d* (days)	$A_{C,Sb}$ = 0.34<br>(BCI: 0.24, 0.57)<br>$A_{G,Sb}$ = 0.012<br>(BCI: 0.0083, 0.018)<br>$A_{S,Sb}$ = 0.012<br>(BCI: 0.0084, 0.030)	Estimated from data
$\lambda_{d,Sb}$	Mean daily egg shedding by *S. bovis* worm pair in host *d* in the absence of density-dependent effects	$\lambda_{C,Sb}$= 218<br>(BCI: 189, 251)<br>$\lambda_{G,Sb}$= 196<br>(BCI: 161, 236)<br>$\lambda_{S,Sb}$= 214<br>(BCI: 176, 258)	Estimated from data
**Model Variables: Definitions**			
$m_{d,j}$	Mean number of worms genotype *j* in host population *d* (Estimation at stability, Table 1 main text)		
$\psi_{d,j}$	Mean number of worm pairs of all combinations in host population *d* which contribute to environmental pool *j*		
L_H	Component of larval pool contributed to by human population; baseline estimate from data (*1*) $\hat{L}_H$=0.84		
L_{Sb}	Component of larval pool contributed to by livestock population; baseline estimate from data (*1*) $\hat{L}_{Sb}$=0.16		

Table S2. Parameters and variables for Haematobium group dynamic transmission model; Definitions, values and sources of estimation. BCI represent 95% Bayesian credible intervals.

Species	Test	Sensitivity Mean (S.D)	Beta coefficients		Source
			β	α	
Small ruminants	KK	0.23 (0.05)	16.06	53.78	(*1*)
	MHT	0.42 (0.06)	28	38.67	
Cattle	KK	0.31 (0.08)	10.05	22.37	
	MHT	0.78 (0.08)	20.13	5.68	

Table S3. Diagnostic test sensitivity priors for Beta distribution used in estimations of parameters in livestock populations: duplicate Kato-Katz (KK), Miracidia hatching technique (MHT), urine filtration and cercarial shedding.

	Miracidia/cercaria Genotype					
	S. haematobium	*Hybrid 1*	*Hybrid 2 Backcross*	*Hybrid2 F1a*	*Hybrid 3 Backcross*	*Hybrid 3 F1b*
**Observed percentage of miracidia analysed[a], N=749**	73.03	24.97	0.53	0.27	1.07	0.13
**Observed percentage of cercariae analysed[b], N=426**	71.59	24.41	0.47	0	3.29	0.24
**Estimated percentage of all miracidia shed in human urine[a] (weighted according to egg shedding by individual host)**	74.50	23.63	0.27	0.08	1.31	0.22
**Number of infected snails shedding genotype**	60	25	2	0	3	1
**Observed percentage of infected snails shedding genotype[c] (95% CI) N=78**	76.92 (66-85.71)	32.05 (21.93-43.58)	2.56 (0.31-8.96)	0 (0-4.62)	3.85 (0.8-10.83)	1.28 (0.03-6.93)
**Estimated proportion of cercarial pool[b] (weighted according to proportion of cercarie of each genotype being shed in co-infected snails)**	72.54	22.85	0.49	0	2.8	1.31
**Estimated mean percentage of worm pairs from Bayesian model (Median, 95% BCI)**	71.99 (69.09, 74.90)	26.40 (23.55, 29.29)	0.29 (0.14,0.53)	0.17 (0.07,0.34)	0.83 (0.47,1.24)	0.26 (0.11, 0.53)

[a] Richard Toll and LDG SAC survey 2016 (*19*)

[b] All Richard Toll and LDG malacology surveys 2015-2018; haematobium group genotypes of human origin (*S. mansoni* and *S. bovis* excluded) (*1*)

[c] Total number of snails infected with genotypes being shed by the human population (snails shedding only *S. bovis* and *S. mansoni* excluded here).

Table S4. Summary of observed miracidia and cercariae genotypes from the human population: Estimated proportion of pool of infectious material derived from human hosts.

CATTLE ABATTOIR: n=19							
**Relationship**	**Parameter**	**Priors**	**Median (95% BCI)**	**Mean (S.D)**	**Deviance**	**Penalty**	**DIC**
Power relationship proportional to total worms	$a_{C,Sb}$	Unif(0.01,100)	0.33 (0.23,0.47)	0.34 (0.06)	883.8	22.87	906.6
	b_C	Unif(-10,0)	-0.55 (-0.62,-0.49)	-0.56 (0.03)			
Power relationship proportional to total couples	$a_{C,Sb}$	Unif(0.01,100)	0.21 (0.16, 0.27)	0.21 (0.03)	903.5	22.16	925.6
	b_C	Unif(-10,0)	-0.57 (-0.63,-0.5)	-0.57 (0.03)			
Exponential relationship proportional to total worms	$a_{C,Sb}$	Unif(0.01,100)	0.053 (0.045,0.062)	0.053 (0.004)	809	22.88	831.9
	b_C	Unif(-10,0)	-0.0033 (-0.0037, -0.0029)	-0.0033 ($2x10^{-4}$)			
Exponential relationship proportional to total couples	$a_{C,Sb}$	Unif(0.01,100)	0.053 (0.045,0.062)	0.053 (0.004)	826.5	22.70	849.5
	b_C	Unif(-10,0)	-0.0085 (-0.0097, -0.0074)	-0.0085 ($6x10^{-4}$)			
**SMALL RUMINANTS ABATTOIR: n=10**							
**Relationship**	**Parameter**	**Priors**	**Median (95% BCI)**	**Mean (S.D)**	**Deviance**	**Penalty**	**DIC**
Power relationship proportional to total worms	$a_{d,Sb}$	Unif(0.01,100)	0.95 (0.81,1.12)	0.95 (0.08)	174.8	11.96	186.7
	b_d	Unif(-10,0)	-0.0075 (-0.039, -0.0028)	-0.011 (0.01)			
Power relationship proportional to total couples	$a_{d,Sb}$	Unif(0.01,100)	0.94 (0.81,1.10)	0.94 (0.08)	174.7	11.95	186.7
	b_d	Unif(-10,0)	-0.0073 (-0.037, $-2.72x10^{-4}$)	-0.01 (0.01)			
Exponential relationship proportional to total worms	$a_{d,Sb}$	Unif(0.01,100)	0.93 (0.8,1.08)	0.93 (0.07)	174.8	11.99	186.8
	b_d	Unif(-10,0)	0.00024 (-0.0012,$1x10^{-5}$)	-0.00033 ($3x10^{-4}$)			
Exponential relationship proportional to total couples	$a_{d,Sb}$	Unif(0.01,100)	0.93 (0.80,1.08)	0.933 (0.07)	174.8	11.97	186.7
	b_d	Unif(-10,0)	-0.00051 ($-2.4x10^{-4}$, $-2x10^{-5}$)	-0.007 ($7x10^{-4}$)			

Table S5 Median posterior values (with 95% Bayesian Credible intervals, BCI) and mean posterior values (with standard deviation, S.D) for estimating fecundity ($a_{d,Sb}$) and density-dependent parameters (b_d) for *Schistosoma bovis* infected cattle (n=19) and small ruminants (n=10). Output from four models with alternative formulation of the density-dependent relationship between worm burden and fecundity.

CATTLE LIVE ANIMAL: n=70			
**Parameter**	**Priors**	**Median (95% BCI)**	**Mean (S.D)**
$a_{C,Sb}$	Gamma(151.92,2866.4)	0.10 (0.09,0.11)	0.10 (0.004)
b_C	Norm(-0.003,2.2x10^{-4})	-0.0029 (-0.003,-0.0029)	-0.0029 (2x10^{-4})
κ_C	Unif(0.01,10)	0.44 (0.32,0.61)	0.45 (0.07)
$m_{C,Sb}$	Unif(1,500)	46.41 (31.83,72.03)	47.87 (10.2)
**GOAT LIVE ANIMAL: n=63**			
**Parameter**	**Priors**	**Median (95% BCI)**	**Mean (S.D)**
$a_{G,Sb}$	Gamma(171.57, 167.11)	0.87 (0.74,1.01)	0.87 (0.07)
b_G	Uni(-0.0012, 1 x10^{-5})	-6.2x10^{-4} (-0.0017, 4 x10^{-5}5)	-6.1x10^{-4} (3x10^{-4})
κ_G	Unif(0.001,10)	0.48 (0.12, 0.97)	0.51 (0.24)
$m_{G,Sb}$	Unif(1,100)	1.11 (1,1.68)	1.16 (0.19)
**SHEEP LIVE ANIMAL: n=68**			
**Parameter**	**Priors**	**Median (95% BCI)**	**Mean (S.D)**
$a_{S,Sb}$	Gamma(171.57, 167.11)	0.95 (0.81,1.10)	0.95 (0.07)
b_S	Unif(-0.0012, 1 x10^{-5})	-6.1x10^{-4} (-0.0012,4x10^{-5})	-6.1x10^{-4} (3x10^{-4})
κ_S	Unif(0.001,10)	0.14 (0.03,0.66)	0.18 (0.16)
$m_{S,Sb}$	Unif(1,100)	1.18 (1.01,5.34)	1.56 (1.25)

Table S6. Median posterior values (with 95% Bayesian Credible intervals, BCI) and mean posterior values (with standard deviation, S.D) for estimation of fecundity ($a_{d,Sb}$), density-dependence (b_d), mean worm burden ($\boldsymbol{m_{d,Sb}}$) and worm dispersion ($\boldsymbol{\kappa_d}$) for cattle, goat and sheep populations.

SI References

1. E. Léger, A. Borlase, C. B. Fall, N. D. Diouf, S. D. Diop, L. Yasenev, S. Catalano, C. T. Thiam, A. Ndiaye, A. Emery, A. Morrell, M. Rabone, M. Ndao, B. Faye, D. Rollinson, J. W. Rudge, M. Sène, J. P. Webster, Prevalence and distribution of schistosomiasis in human, livestock, and snail populations in northern Senegal: a One Health epidemiological study of a multi-host system. *Lancet. Planet. Heal.* **4**, e330–e342 (2020).
2. N. Katz, A. Chaves, J. Pellegrino, A simple device for quantitative stool thick-smear technique in Schistosomiasis mansoni. *Rev. Inst. Med. Trop. Sao Paulo*. **14**, 397–400 (1972).
3. R. M. May, Togetherness among Schistosomes: its effects on the dynamics of the infection. *Math. Biosci.* **35**, 301–343 (1977).
4. R. M. Anderson, R. M. May, Helminth infections of humans: mathematical models, population dynamics, and control. *Adv Parasitol*. **24** (1985), doi:10.1016/S0065-308X(08)60561-8.
5. R. M. May, M. E. Woolhouse, Biased sex ratios and parasite mating probabilities. *Parasitology*. **107 Pt 3**, 287–295 (1993).
6. S. Morand, V. R. Southgate, J. Jourdane, A model to explain the replacement of Schistosoma intercalatum by *Schistosoma haematobium* and the hybrid *S. intercalatum* x *S. haematobium* in areas of sympatry. *Parasitology*. **124**, 401–8 (2002).
7. World Health Organisation Preventative chemotherapy databank, (available at www.who.int/neglected_diseases/preventive_chemotherapy/sch/en).
8. J. Zwang, P. L. Olliaro, Clinical Efficacy and Tolerability of Praziquantel for Intestinal and Urinary Schistosomiasis—A Meta-analysis of Comparative and Non-comparative Clinical Trials. *PLoS Negl. Trop. Dis.* (2014), doi:10.1371/journal.pntd.0003286.
9. S. Trouve, P. Sasal, J. Jourdane, F. Renaud, S. Morand, The evolution of life-history traits in parasitic and free-living platyhelminthes: a new perspective. *Oecologia*. **115**, 370–378 (1998).
10. G. Medley, R. M. Anderson, Density-dependent fecundity in schistosoma mansoni infections in man. *Trans. R. Soc. Trop. Med. Hyg.* **79**, 532–534 (1985).
11. J. Boissier, H. Mone, Experimental observations on the sex ratio of adult Schistosoma mansoni, with comments on the natural male bias. *Parasitology* (2000), doi:10.1017/S0031182099006393.

12. S. van Buuren, C. Groothuis-Oudshoorn, MICE: Multivariate Imputation by Chained Equations in R. *J. Stat. Softw.* **45** (2011), doi:10.18637/jss.v045.i03.
13. A. Hall, C. Holland, Geographical variation in Ascaris lumbricoides fecundity and its implications for helminth control. *Parasitol. Today*. **16**, 540–544 (2000).
14. T. S. Churcher, N. M. Ferguson, M. G. Basanez, Density dependence and overdispersion in the transmission of helminth parasites. *Parasitology*. **131**, 121–132 (2005).
15. C. M. Gower, F. Gehre, S. R. Marques, P. H. L. Lamberton, N. J. Lwambo, J. P. Webster, Phenotypic and genotypic monitoring of Schistosoma mansoni in Tanzanian schoolchildren five years into a preventative chemotherapy national control programme. *Parasit. Vectors*. **10**, 593 (2017).
16. R. M. Anderson, R. M. May, Infectious Diseases of Humans: Dynamics and Control. *(Oxford Univ. Press. Oxford, UK)* (1991).
17. J. E. Truscott, D. Gurarie, R. Alsallaq, J. Toor, N. Yoon, S. H. Farrell, H. C. Turner, A. E. Phillips, H. O. Aurelio, J. Ferro, C. H. King, R. M. Anderson, A comparison of two mathematical models of the impact of mass drug administration on the transmission and control of schistosomiasis. *Epidemics*. **18**, 29–37 (2017).
18. R. M. Anderson, H. C. Turner, S. H. Farrell, J. Yang, J. E. Truscott, What is required in terms of mass drug administration to interrupt the transmission of schistosome parasites in regions of endemic infection? *Parasit. Vectors*. **8**, 553 (2015).
19. D. Gurarie, N. Yoon, E. Li, M. Ndeffo-Mbah, D. Durham, A. E. Phillips, H. O. Aurelio, J. Ferro, A. P. Galvani, C. H. King, Modelling control of Schistosoma haematobium infection: predictions of the long-term impact of mass drug administration in Africa. *Parasit. Vectors*. **8**, 529 (2015).
20. Z. Feng, A. Eppert, F. A. Milner, D. J. Minchella, Estimation of parameters governing the transmission dynamics of schistosomes. *Appl. Math. Lett.* **17**, 1105–1112 (2004).
21. A. Gelman, D. B. Rubin, Inference from Iterative Simulation Using Multiple Sequences. *Stat. Sci.* **7**, 457–472 (1992).
22. S. P. Brooks, A. Gelman, General Methods for Monitoring Convergence of Iterative Simulations. *J. Comput. Graph. Stat.* **7**, 434–455 (1998).
23. J. K. Kruschke, *Doing Bayesian data analysis: A tutorial with R, JAGS, and Stan, second edition* (2014).
24. M. Plummer, rjags: Bayesian graphical models using MCMC. (2018).
25. J. Vercruysse, V. R. Southgate, D. Rollinson, The epidemiology of human and animal schistosomiasis in the Senegal River Basin. *Acta Trop.* **42**, 249–259 (1985).

26. F. G. Marill, Diffusion de la bilharziose chez les bovins, ovins et caprins en Mauritanie et dans la vallée du Sénégal. *Bull. Acad. Natl. Med.* **145**, 147–150 (1961).
27. R. M. Anderson, J. Crombie, Experimental studies of age-prevalence curves for *Schistosoma mansoni* infections in populations of *Biomphalaria glabrata*. *Parasitology*. **89 (Pt 1)**, 79–105 (1984).
28. G. M. Williams, A. C. Sleigh, Y. Li, Z. Feng, G. M. Davis, H. Chen, A. G. P. Ross, R. Bergquist, D. P. McManus, Mathematical modelling of schistosomiasis japonica: comparison of control strategies in the People's Republic of China. *Acta Trop.* **82**, 253–262 (2002).
29. J. W. Rudge, J. P. Webster, D.-B. Lu, T.-P. Wang, G.-R. Fang, M.-G. Basanez, Identifying host species driving transmission of schistosomiasis japonica, a multihost parasite system, in China. *Proc. Natl. Acad. Sci.* **110**, 11457–11462 (2013).
30. T.-P. P. Wang, M. Vang Johansen, S.-Q. Q. Zhang, F.-F. F. Wang, W.-D. D. Wu, G.-H. H. Zhang, X.-P. P. Pan, Y. Ju, N. Ornbjerg, Transmission of *Schistosoma japonicum* by humans and domestic animals in the Yangtze River valley, Anhui province, China. *Acta Trop.* **96**, 198–204 (2005).
31. M. G. Roberts, J. A. P. Heesterbeek, A new method for estimating the effort required to control an infectious disease. *Proceedings. Biol. Sci.* **270**, 1359–1364 (2003).
32. A. Dobson, Population Dynamics of Pathogens with Multiple Host Species. *Source Am. Nat.* **164**, 64–78 (2004).
33. C. M. Gower, L. Vince, J. P. Webster, Should we be treating animal schistosomiasis in Africa? The need for a One Health economic evaluation of schistosomiasis control in people and their livestock. *Trans. R. Soc. Trop. Med. Hyg.* **111**, 244–247 (2017).
34. H. O. Bushara, B. Y. Majid, A. A. Majid, I. Khitma, A. A. Gameel, E. A. Karib, M. F. Hussein, M. G. Taylor, Observations on cattle schistosomiasis in the Sudan, a study in comparative medicine. V. The effect of praziquantel therapy on naturally acquired resistance to Schistosoma bovis. *Am. J. Trop. Med. Hyg.* **32**, 1370–1374 (1983).
35. PEPAM: Programme d'eau potable et d'assainissement du Millénaire:, (available at www.pepam.gouv.sn).
36. J. Fransen, J. De Bont, J. Vercruysse, D. Van Aken, V. R. Southgate, D. Rollinson, Pathology of natural infections of Schistosoma spindale Montgomery, 1906, in cattle. *J. Comp. Pathol.* **103**, 447–455 (1990).
37. R. T. Sani, G. E. Jokhtan, Manure Production by Yankasa Sheep Grazing Natural Pastures in Northern Guinea Savannah Zone of Nigeria. *J. Anim. Prod. Res.* **27** (2015).

38. C. U. Osuhor, J. P. Alawa, G. N. Akpa, Research note: Manure production by goats grazing native pasture in Nigeria. *Trop. Grasslands*. **36**, 123–125 (2002).

39. N. K. R. Musimba, M. L. Galyean, J. L. Holechek, R. D. Pieper, Ytterbium-labeled Forage as a Marker for Estimation of Cattle Fecal Output. *J. Range Manag.* **40**, 418–421 (1987).

40. F. Mahler, E. Schlecht, M. Sangare, K. Becker, Granulated polyamide as external marker to estimate total faecal excretion of grazing cattle in extensive management systems. *Br. J. Nutr.* **78**, 785–803 (1997).
